# Metabolomic and transcriptomic insights into how cotton fiber transitions to secondary wall synthesis, represses lignification, and prolongs elongation

**DOI:** 10.1186/s12864-015-1708-9

**Published:** 2015-06-27

**Authors:** John R. Tuttle, Gyoungju Nah, Mary V. Duke, Danny C. Alexander, Xueying Guan, Qingxin Song, Z. Jeffrey Chen, Brian E. Scheffler, Candace H. Haigler

**Affiliations:** Department of Crop Science, North Carolina State University, Raleigh, NC 27695 USA; Institute for Cellular and Molecular Biology and Center for Computational Biology and Bioinformatics, The University of Texas at Austin, Austin, TX 78712 USA; USDA ARS Genomics and Bioinformatics Research Unit, Stoneville, MS 38776 USA; Metabolon Inc, Durham, NC 27713 USA; Department of Plant and Microbial Biology, North Carolina State University, Raleigh, NC 27695 USA

**Keywords:** Ascorbate, Cell elongation, Cell wall synthesis, Cotton fiber development, *Gossypium*, Lignification, Reactive oxygen species, Metabolomics, RNA Seq transcriptomics

## Abstract

**Background:**

The morphogenesis of single-celled cotton fiber includes extreme elongation and staged cell wall differentiation. Designing strategies for improving cotton fiber for textiles and other uses relies on uncovering the related regulatory mechanisms. In this research we compared the transcriptomes and metabolomes of two *Gossypium* genotypes, *Gossypium barbadense* cv Phytogen 800 and *G. hirsutum* cv Deltapine 90. When grown in parallel, the two types of fiber developed similarly except for prolonged fiber elongation in the *G. barbadense* cultivar. The data were collected from isolated fibers between 10 to 28 days post anthesis (DPA) representing: primary wall synthesis to support elongation; transitional cell wall remodeling; and secondary wall cellulose synthesis, which was accompanied by continuing elongation only in *G. barbadense* fiber.

**Results:**

Of 206 identified fiber metabolites, 205 were held in common between the two genotypes. Approximately 38,000 transcripts were expressed in the fiber of each genotype, and these were mapped to the reference set and interpreted by homology to known genes. The developmental changes in the transcriptomes and the metabolomes were compared within and across genotypes with several novel implications. Transitional cell wall remodeling is a distinct stable developmental stage lasting at least four days (18 to 21 DPA). Expression of selected cell wall related transcripts was similar between genotypes, but cellulose synthase gene expression patterns were more complex than expected. Lignification was transcriptionally repressed in both genotypes. Oxidative stress was lower in the fiber of *G. barbadense* cv Phytogen 800 as compared to *G. hirsutum* cv Deltapine 90. Correspondingly, the *G. barbadense* cultivar had enhanced capacity for management of reactive oxygen species during its prolonged elongation period, as indicated by a 138-fold increase in ascorbate concentration at 28 DPA.

**Conclusions:**

The parallel data on deep-sequencing transcriptomics and non-targeted metabolomics for two genotypes of single-celled cotton fiber showed that a discrete developmental stage of transitional cell wall remodeling occurs before secondary wall cellulose synthesis begins. The data showed how lignification can be transcriptionally repressed during secondary cell wall synthesis, and they implicated enhanced capacity to manage reactive oxygen species through the ascorbate-glutathione cycle as a positive contributor to fiber length.

**Electronic supplementary material:**

The online version of this article (doi:10.1186/s12864-015-1708-9) contains supplementary material, which is available to authorized users.

## Background

Cotton fibers are single cells that elongate from the epidermis of the cotton ovule beginning near the day of anthesis. Cotton fiber morphogenesis includes initiation, elongation via primary cell wall synthesis, transitional cell wall remodeling, secondary cell wall (SCW) synthesis, and maturation. The fibers used for yarn and textile manufacturing contain 90 to 95 % cellulose in a secondary wall surrounded by a thin cuticulated primary wall. Cell wall differentiation is a major driver of cotton fiber morphogenesis and the final quality of cotton fiber [1]. The staged development of this single cell offers unprecedented opportunities to use comprehensive transcriptomics and metabolomics to gain insight into the molecular and biochemical regulation of cell elongation, cell wall synthesis, and fiber quality.

To explore the mechanisms regulating cotton fiber morphogenesis and quality, we analyzed two genotypes of modern commercial cotton with higher and lower fiber quality. Specifically, we analyzed a modern cultivar from each of the two allotetraploid (AD genome) species, *Gossypium hirsutum* (*Gh;* AD_1_) and *Gossypium barbadense* (*Gb;* AD_2_). The commercial allotetraploids derived from a single polyploidization event about 1 to 2 million years ago involving two diploid ancestors similar to extant *G. raimondii* (D genome) and *G. arboreum/G. herbaceum* (A genome). Both A and D genome diploid ancestral cottons had seed hairs (cotton fiber), with those in extant wild A genome species most closely resembling AD commercial fiber [2]. Independent natural selection and domestication trajectories from one ancestral allotetraploid led to modern *Gh* and *Gb* cotton, which has superior fiber properties [3, 4]. *Gh* (or ‘Upland’ cotton) is predominantly grown for its high yield in more varied environments, whereas *Gb* (or ‘Pima’ cotton) is grown to provide longer, stronger, and finer fiber for the manufacture of higher quality textiles [5].

Several studies have compared paired accessions of *Gb* and *Gh* fibers to look for the controls of cotton fiber quality. These studies have uncovered differences related to the biosynthesis of flavonoids, lignin-related phenolics, cell wall molecules, and proteins, as well as protein degradation [6–9]. Considered together, these comparisons included variable times between 5 to 34 DPA for fiber from different cultivars and growing conditions, both of which can shift the timing of fiber development and comparative fiber quality. In addition, these prior transcriptomic comparisons were based on 454 sequencing and microarray technology, creating a need for thorough comparative analysis using ultra-deep Illumina sequencing. Limited metabolite comparisons had been done before, showing higher concentrations of flavonoids including naringenin and dihydrokaempherol in 5 and 10 DPA *Gh* fiber as compared to *Gb* fiber [9].

Here we report transcriptomics based on Illumina deep sequencing and non-targeted metabolomics on the same fiber samples of two genotypes at 10, 15, 18, 21, and 28 DPA. The cultivars analyzed here were *Gh* cv Deltapine 90 and *Gb* cv Phytogen 800. They exhibit the expected fiber quality differences when grown in parallel under controlled growth conditions and have well characterized fiber developmental profiles [10]. Specifically, the fibers of both genotypes had similar: (a) rates of elongation between 10 to 22 DPA; (b) timing of transitional cell wall remodeling (17 to 21 DPA); and (c) timing of the onset (22 DPA) and termination (45 DPA) of SCW thickening. At 28 DPA, SCW cellulose synthesis was ongoing in both fiber genotypes, but late elongation was continuing only in *Gb* fiber. Using this background knowledge, we were able to compare the data from *Gh* and *Gb* fiber at each DPA in a highly similar physiological context while also looking for potential regulators of the sustained elongation of *Gb* fiber. Below, we briefly review the context for the major results.

### Molecular regulation of cell wall synthesis

Cell walls compose our most abundant renewable biomaterials, so the regulation of their synthesis is of general interest. The staged development of single-celled cotton fiber can reveal regulatory mechanisms with less ambiguity than for other cell types that are differentiating within complex tissues. Elongating cotton fiber has typical primary walls [11]. Near the beginning of secondary wall synthesis, a thin ‘winding’ cell wall layer is deposited that contributes substantially to cotton fiber strength [12–14]. Most fiber thickening depends on the synthesis of a SCW composed of 90–95 % cellulose, and this occurs through use of a genetic and biochemical program similar to sclerenchyma cells [15]. As shown by numerous studies, genes encoding cell wall polymer synthases, structural proteins, and regulators are abundant within the cotton fiber transcriptome [6, 8, 16]. Here we focused our comparative analyses on genes encoding: (a) cellulose synthases (CESAs), which support the synthesis of strong cellulose fibrils within both primary and secondary walls [17]; (b) cellulose-synthase-like enzymes, which generate the backbones of diverse cell wall matrix polymers [18]; (c) transcription factors that regulate the onset of SCW synthesis in sclerenchyma cells [19, 20]; and (d) enzymes within the flavonoid and lignin biosynthetic pathways [21, 22].

### Defining the transition between primary and secondary cell wall synthesis

Transitional cell wall layers between the primary and secondary wall are increasingly recognized as important for determining the mechanical properties of cotton fiber and wood [13, 14, 23]. A ‘transition stage’ occurs at the onset of cotton fiber secondary wall synthesis, as reviewed previously [1]. Metabolic and cellular changes at this time include a transient reduction in fiber respiration rate and lowering of glucose and fructose concentrations [24]. The ‘winding’ cell wall layer is deposited with a polymer composition similar to the primary wall. This thin transitional layer is synthesized while the content of crystalline cellulose in the fiber cell wall increases to about 25 % and both microtubules and cellulose fibrils shift to shallow helical orientations [11, 12]. The cotton fiber middle lamella (CFML), which previously joined the fibers together during earlier elongation, is degraded in parallel with changes in the size and composition of cell wall matrix polysaccharides [10, 11, 25–28].

### Management of reactive oxygen species

Reactive oxygen species (ROS) including singlet oxygen, superoxide, hydrogen peroxide (H_2_O_2_), and hydroxyl radicals are generated as normal byproducts of aerobic respiration and also act as signals in development, defense, and stress responses [29]. ROS have several roles in cotton fiber development. Fiber initiation was increased by exogenous H_2_O_2_ in a fuzzless mutant [30]. Early cotton fiber elongation requires high levels of superoxide [31]. Similarly, adding H_2_O_2_ to 5 DPA cotton ovule cultures stimulated fiber elongation, coincident with the potential for maintaining ROS homeostasis through the increased expression and activity of cytosolic ascorbate peroxidase (APX) [32]. Over-expressing chloroplast-targeted pea *APX* was associated with increased fiber length in one *Gh* cotton line compared to its segregating null line in field experiments, but there was no difference compared to the wild-type line and no rationale provided for an effect on fiber [33]. The transition to SCW synthesis depends on a spike in H_2_O_2_ [34, 35], with potential downstream effects including the dimerization of cellulose synthases [36]. Several transcriptomic studies have implicated ROS management in the improvement of fiber quality during domestication [37–39], and relevant enzymes have been identified through proteomics [40]. For example, certain isoforms of dehydroascorbate reductase (DHAR) and APX were more abundant in domesticated *Gh* with longer fiber compared to its wild progenitor [41].

Despite their developmental roles, prolonged exposure to ROS in high concentrations can damage cells, causing oxidation of lipids and proteins and inducing programmed cell death in extreme cases [29, 42]. Sulfur-containing cysteine or methionine residues are commonly reversibly oxidized, and irreversible carbonyl derivatives of other amino acids can be produced. Either process can change protein conformation and function, along with activation of proteolysis or autophagy to remove damaged proteins [42–44]. Therefore, cells use chemical and enzymatic processes to control ROS concentrations. Common ROS-scavenging chemical reductants include: ascorbate (Vitamin C), glutathione, tocopherol, carotenoids, and phenolic compounds such as flavonoids [29, 45, 46]. The ascorbate-glutathione cycle is a major contributor to enzymatic ROS scavenging in plants [29], and transgenic experiments support similar effects in cotton fiber. Although the mechanism was not completely understood due to targeting of transgenes to the plastid, the fineness (mass per unit length) and bundle strength of *Gh* cotton fiber were increased through over-expression of *APX* or glutathione reductase (*GSR*, also abbreviated *GR*) [33]. Differences in ROS management in *Gh* and *Gb* cotton fibers will be discussed later.

### Overview of the experimental approach and major results

We analyzed two cultivars of *Gb* and *Gh* fiber grown in parallel at 10 and 15 DPA (during rapid primary wall synthesis supporting elongation), 18 and 21 DPA (during transitional cell wall remodeling), and 28 DPA (during SCW cellulose synthesis, plus the last stage of elongation in *Gb* fiber). The metabolome was analyzed in fiber extracts using ion-optimized mass spectrometry and gas chromatography to reveal 206 total compounds, with 205 of them present in the fiber of both genotypes, and no unidentified compounds. For transcriptomic analysis, more than 2 billion high quality, 100-bp, paired-end reads were generated on an Illumina HiSeq2000 and mapped to a reference set derived from D-genome diploid cotton, *G. raimondii* (*Gr*; [47]) and expressed sequence tags from A-genome *G. arboreum* (*Ga*; [48]).

The transcriptomes and metabolomes were compared within the two genotypes across DPA and between genotypes at the same DPA to reach several novel conclusions. Previously, we highlighted how the well-characterized fiber development profiles in the two cultivars analyzed here allow us to meaningfully interpret the data. Major conclusions include: defining a discrete stage of transitional cell wall remodeling; showing how cotton fiber has modified an ancient transcription factor network regulating SCW synthesis to repress lignification; and providing a biochemical pathway perspective on ROS management in cotton fiber that reveals differences leading to less oxidative stress in a longer form of cotton fiber. This difference in ROS management, along with sustained transcription of elongation-associated genes, correlated with the prolonged elongation period of *Gb* cv Phytogen 800 fiber as compared to *Gh* cv Deltapine 90 fiber. Collectively, the data provide clues about future testable strategies for increasing fiber length in short-fibered *Gossypium* genotypes. We reanalyzed the microarray data from another pair of *Gh* and *Gb* cultivars [7], finding consistency with our data. Nonetheless, in this paper we use the abbreviations *Gh* and *Gb* for the concise indication of the genotypes studied without implying any generalization of the results to other accessions in the two species. We anticipate that the data will stimulate further tests of these findings in cotton diversity panels and through various biotechnology and breeding strategies.

## Results and discussion

### Overall transcriptomic and metabolic outcomes

Illumina sequencing of the transcriptomes of 10, 15, 18, 21, and 28 DPA *Gh* and *Gb* fiber yielded 2,015,378,704 high quality, multiplexed, 100-bp, paired end reads with quality scores ≥ 33 (deposited at NCBI Sequence Read Archive (SRA); study project number SRP049330). Nearly all (98 %) of the 77,267 transcripts in the *Gr* genome-scale reference set were detected. Not all of these transcripts are likely to be biologically relevant to cotton fiber development, so we analyzed only 41,566 total unique transcripts from the fiber of both genotypes with reads per kilobase per million reads mapped (RPKM) values ≥ 2. These represented 42 % of the total detected transcripts (Additional files 1 and 2). The number of transcripts expressed in *Gb* or *Gh* fiber (37,936 or 38,337) was nearly the same.

Fiber metabolites from the same samples were profiled by gas and liquid chromatography followed by mass spectrometry and identified by reference to approximately 3000 standards. All signals could be matched to standards and 205 of 206 total metabolites were identified in the fiber of both genotypes. One dipeptide (gamma-glutamylglutamate) was identified only in *Gb* fiber. Of the 206 total metabolites, 152 were curated in the KEGG database (www.genome.jp/kegg/) (Additional file 3).

Excluding seven broad pathways, between 1–20 metabolites fell into 73 more specific KEGG pathways (Additional file 4). Fifteen pathways (inclusive of 69 metabolites) were overrepresented in cotton fiber based on the number of metabolites in the pathway (Table 1), reflecting a strong demand for energy, amino acids, and carbohydrates. The sugar-related pathway, ‘galactose metabolism’ was populated by 11 metabolites: glucose, glucose-6-phosphate, UDP-glucose, fructose, sucrose, UDP-galactose, galactinol, glycerol, myo-inositol, raffinose, and stachyose. These represent precursors of carbohydrate and cell wall synthesis as well as end products (raffinose and stachyose oligosaccharides). The ‘glutathione metabolism’ pathway related to amino acid metabolism and ROS management was populated by 10 metabolites: glutamate, glycine, reduced and oxidized glutathione, ascorbate, cysteine, putrescine, spermidine, 5-oxoproline, and dehydroascorbate. These key aspects of cotton fiber metabolism will be discussed further below.

**Table 1:** Overrepresented KEGG pathways in cotton fiber implicated by metabolites or enzyme-encoding transcripts. Entries reflect the combined data from *Gh* and *Gb* fiber

Metabolome	Transcriptome
*Alanine, aspartate and glutamate metabolism*	*Alanine, aspartate and glutamate metabolism*
*Aminoacyl-tRNA biosynthesis*	*Aminoacyl-tRNA biosynthesis*
Ascorbate and aldarate metabolism	Carbon fixation in photosynthetic organisms
beta-Alanine metabolism	*Citrate cycle (TCA cycle)*
Butanoate metabolism	Cysteine and methionine metabolism
*Citrate cycle (TCA cycle)*	Flavonoid biosynthesis
Cyanoamino acid metabolism	Glycerolipid metabolism
Galactose metabolism	Glycolysis/Gluconeogenesis
Glutathione metabolism	One carbon pool by folate
Glycine, serine and threonine metabolism	Other glycan degradation
Glyoxylate and dicarboxylate metabolism	*Oxidative phosphorylation*
*Oxidative phosphorylation*	Phenylalanine, tyrosine, tryptophan biosynthesis
Pantothenate and CoA biosynthesis	Phosphatidylinositol signaling system
Taurine and hypotaurine metabolism	Purine metabolism
*Valine, leucine and isoleucine biosynthesis*	Starch and sucrose metabolism
	*Valine, leucine and isoleucine biosynthesis*

*Italics* indicate overrepresented pathways implicated by both metabolites and transcripts. Overrepresentation of pathways (*p* < 0.02, q < 0.09) was determined by Fisher’s exact test followed by Benjamini-Hochberg correction of the *p*-values (see Additional files 4 and 7)

The 41,566 transcripts with RPKM ≥ 2 were analyzed with Blast2GO to identify homologs of 913 unique known enzymes (1E^−10^, Additional file 5), of which 692 mapped to KEGG pathways (Additional file 6). As inferred from transcripts, sixteen pathways were potentially overrepresented (Table 1, Additional file 7), and five of these related to energy and amino acids were also overrepresented in the metabolome: alanine aspartate and glutamate metabolism; aminoacyl-tRNA biosynthesis; citrate (TCA) cycle; oxidative phosphorylation; and valine, leucine, and isoleucine biosynthesis. Other pathways overrepresented by transcripts emphasize the importance of carbohydrates in cotton fiber, as well as pointing to more specific processes such as flavonoid biosynthesis (also potentially related to ROS scavenging), glycerolipid metabolism, and phosphatidylinositol signaling.

### Overview of changes in the transcriptome in 10 to 28 DPA cotton fiber

**Fig. 1:**
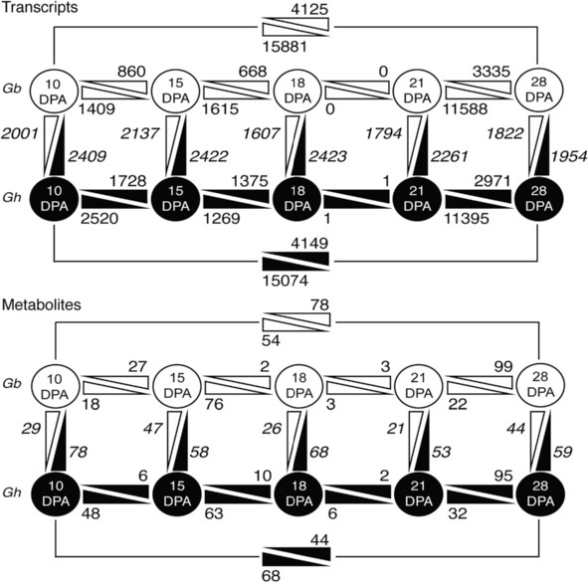
Quantitative changes in the transcriptome and metabolome within and between *Gb* and *Gh* fibers. Upper panel: the numbers of transcripts with at least 2 fold up- or downregulation (q ≤ 0.001). Lower panel: the numbers of metabolites with different concentrations (p ≤ 0.05). Each panel shows data for *Gb* fiber (top, white) and *Gh* fiber (bottom, black). On the horizontal axis, numbers are placed nearest the DPA when that number of transcripts or metabolites was higher. On the vertical axis, italicized numbers are placed nearest the genotype in which the transcripts or metabolites were higher on each DPA. The comparisons at 10 vs 28 DPA are also shown to highlight the contrast between primary and secondary wall synthesis, a comparison that is most distinct for *Gh* fiber that is no longer elongating at 28 DPA

We analyzed the changes in transcripts across the time-course of fiber development within one genotype and at the same DPA across genotypes (Fig. 1). Notably, about 88 % of the transcripts were expressed at 10 DPA in *Gh* or *Gb* fiber (33,310 or 33,837 transcripts, respectively), with similar expression levels maintained through 21 DPA. Some of these are alternative transcripts from the same locus, and the 10 DPA unique transcripts are homologous to 14,006 or 14,344 (37–38 %) of the 37,505 protein-coding loci in *Gr* D-genome diploid cotton. The largest change in gene expression for the fiber of both genotypes occurred between 21 to 28 DPA, representing the shift to predominantly SCW cellulose synthesis. By reference to 21 DPA, more transcripts were downregulated at 28 DPA (11,395 or 11,588 for *Gh* or *Gb*) than were upregulated (2971 or 3335 for *Gh* or *Gb*). This resulted in 23,834 or 24,297 transcripts expressed in 28 DPA *Gb* or *Gh* fiber (Additional files 1 and 2). Comparing 10 and 28 DPA, representing the dominance of primary vs secondary wall synthesis, similarly showed 15,074–15,881 downregulated transcripts at 28 DPA as compared to 4,125–4,149 upregulated transcripts. The GO terms that were overrepresented by the transcripts of both genotypes at 28 DPA compared to 21 DPA include: SCW biogenesis, cellulose microfibril organization, and numerous carbohydrate terms (sucrose, fructose, xylose, mannose, galactose, starch, trehalose). In addition, the overrepresented GO terms reflected the regulation of a major developmental shift: activation of MAPKK activity, negative regulation of ethylene-mediated signaling pathway, and transmembrane receptor protein serine/threonine kinase signaling pathway (Table 2). The homologous *Gr* gene IDs in each GO category can be found in Additional file 8.

**Table 2:** Overrepresented Gene Ontology biological process terms in 28 DPA vs 21 DPA fiber. Transcripts that had higher expression (fold change ≥2) at 28 vs 21 DPA in both *Gb* and *Gh* fiber were analyzed for over-representation of GO biological process terms (*p* < 0.007, q < 0.05). False discovery rate q-values (FDR) were determined as in Table 1 (see *p*-values in Additional file 8)

GO ID	GO Name	*Gb*-FDR	*Gh*-FDR
GO:0000186	activation of MAPKK activity	1.43E-04	8.60E-05
GO:0010215	cellulose microfibril organization	4.17E-02	3.43E-02
GO:0015996	chlorophyll catabolic process	2.05E-04	5.54E-05
GO:0030206	chondroitin sulfate biosynthetic process	1.06E-05	6.00E-05
GO:0035434	copper ion transmembrane transport	4.01E-05	6.06E-03
GO:0010143	cutin biosynthetic process	8.11E-04	2.30E-05
GO:0006535	cysteine biosynthetic process from serine	2.68E-04	7.06E-05
GO:0006014	D-ribose metabolic process	1.43E-04	1.25E-04
GO:0042732	D-xylose metabolic process	2.25E-03	2.61E-02
GO:0006000	fructose metabolic process	2.82E-03	7.44E-07
GO:0006012	galactose metabolic process	2.49E-05	3.06E-03
GO:0009298	GDP-mannose biosynthetic process	2.23E-02	4.82E-02
GO:0010417	glucuronoxylan biosynthetic process	5.97E-03	2.07E-02
GO:0006424	glutamyl-tRNA aminoacylation	1.67E-03	1.60E-03
GO:0019310	inositol catabolic process	4.01E-05	7.59E-03
GO:0006559	L-phenylalanine catabolic process	2.25E-03	2.02E-03
GO:0007142	male meiosis II	2.07E-02	2.01E-02
GO:0019307	mannose biosynthetic process	5.97E-03	5.70E-03
GO:0009556	microsporogenesis	2.07E-02	4.66E-04
GO:0010105	negative regulation of ethylene mediated signaling pathway	2.06E-02	1.86E-02
GO:0009878	nodule morphogenesis	1.67E-03	1.60E-03
GO:0018106	peptidyl-histidine phosphorylation	8.84E-07	9.75E-05
GO:0009915	phloem sucrose loading	2.07E-02	2.01E-02
GO:0010205	photoinhibition	2.07E-02	2.01E-02
GO:0010043	response to zinc ion	6.40E-05	6.51E-06
GO:0009834	secondary cell wall biogenesis	7.21E-08	5.51E-07
GO:0005982	starch metabolic process	6.95E-07	1.07E-05
GO:0010345	suberin biosynthetic process	2.07E-02	5.70E-03
GO:0005985	sucrose metabolic process	4.88E-10	6.86E-08
GO:0007178	transmembrane receptor protein serine/threonine kinase signaling pathway	8.44E-05	2.37E-05
GO:0005992	trehalose biosynthetic process	4.57E-04	2.16E-04
GO:0006572	tyrosine catabolic process	1.43E-04	1.25E-04

**Fig. 2:**
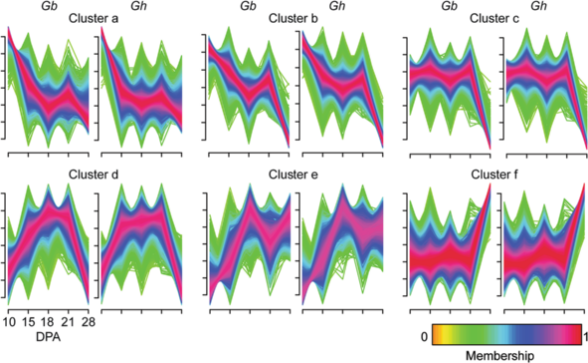
*Gb* and *Gh* fiber showed six similar clusters of gene expression patterns. All transcripts differentially expressed during fiber development (q ≤ 0.001) were clustered. Each x-axis tick corresponds to expression level at 10, 15, 18, 21, or 28 DPA, with RPKM expression values (on the y-axis) standardized to mean = 0 and standard deviation = 1. Transcript patterns in each cluster are color coded by membership value, with higher membership value indicating a higher likelihood that a given transcript belongs in a cluster

Of ~38,000 non-redundant transcripts expressed across all DPA in each fiber genotype, approximately 94 % showed significantly different expression levels on at least one day between 10 to 28 DPA (q ≤0.001), and of these approximately 14 % showed fold change ≥ 2 (Additional files 9 and 10). Six major differential expression patterns were identified by use of Fuzzy C-means clustering [49] (Fig. 2). The color scale indicates the confidence level for cluster membership, with red to magenta indicating ≥ 70 % confidence in cluster assignment. The similarity of clusters for *Gh* and *Gb* fiber corresponds to many similarities in their fiber developmental programs [10]. The number of genes in each cluster ranged from 2,463 (*Gb* cluster e) to 9,370 (*Gb* cluster b), with only 3–9.6 % difference in the number of *Gh* or *Gb* genes in the paired clusters. The gene expression clusters correspond to known aspects of fiber development as interpreted below by reference to selected GO biological processes or genes overrepresented in both fiber genotypes (Additional file 11).

Cluster ‘a-transcripts’ includes genes upregulated at 10 DPA near the beginning of high-rate fiber elongation. Reflecting rapid cell growth and related biosynthetic demands, the overrepresented GO terms include: positive regulation of cell size, reductive tricarboxylic acid cycle, pentose phosphate shunt, fatty acid biosynthesis, and numerous terms related to amino acids. Cluster ‘b-transcripts’ includes genes upregulated at 10 DPA with another lower level of upregulation at 21 DPA. Genes in this cluster may be needed to initiate high-rate primary and secondary wall synthesis, and the overrepresented GO terms include: cellulose microfibril organization; carbon utilization; xylem development; ribosome biogenesis, chaperone mediated protein folding, and regulation of translational initiation. Cluster ‘c-transcripts’ includes genes upregulated at 10 to 21 during fiber elongation before the onset of SCW synthesis. The overrepresented GO terms in both genotypes are less informative for this cluster, but shared individual genes reflect the control of fiber elongation by turgor pressure (cotton genes related to Arabidopsis *PIP2* and Gorai.002G002500.1) and expansin proteins (cotton genes related to *AtEXP8* and Gorai.011G128500.2). Cluster ‘d-transcripts’ includes genes upregulated at 15 to 21 DPA during the initiation and maintenance of transitional cell wall remodeling. The overrepresented GO terms include: detection of ethylene-mediated signaling; cell wall macromolecule catabolic process; (1-3)-beta-D-glucan biosynthetic process; and ROS-related terms. Cluster ‘e-transcripts’ includes genes upregulated at 18 to 28 DPA with slightly lower expression at 21 DPA, during synthesis of the winding layer and the cellulose-rich SCW. The overrepresented GO terms include: early endosome to late endosome transport and protein stabilization, both of which may relate to the transport and function of cell wall biosynthetic enzymes. Cluster ‘f-transcripts’ includes genes upregulated at 28 DPA, and the overrepresented GO terms include: SCW biogenesis, cellulose microfibril organization, and terms related to sucrose, glucan biosynthesis, and vesicle trafficking.

### Differences in the transcriptome between *Gh* and *Gb* fibers

A total of 20,830 transcripts were differentially expressed (fold change ≥ 2) between *Gh* and *Gb* fiber, with approximately 2,000 transcripts showing changed expression at any one DPA as compared to the other genotype (Fig. 1, Additional files 12 and 13). Among these, there were 4,551 or 5,252 non-redundant differentially expressed transcripts in *Gb* or *Gh* fiber, and most of these were uniquely found in one fiber genotype. The transcripts with higher expression in *Gb* fiber mapped to 25 overrepresented GO terms (q ≤ 0.01), including 6 terms associated with protein biosynthesis. In contrast, the transcripts with higher expression in *Gh* fiber mapped to 30 overrepresented GO terms, with 7 terms associated with biotic and abiotic stress responses (Table 3). The homologous *Gr* gene IDs in each GO category can be found in Additional file 14. The biological context of selected differences between genotypes will be discussed further below.

**Table 3:** Overrepresented Gene Ontology biological process terms in *Gb* or *Gh* fiber. Transcripts that had higher expression (fold change ≥2) on at least one DPA in the cross genotype comparison were analyzed for over-representation of GO biological process terms (*p* < 0.006, q < 0.05). FDR q-values were determined as in Table 1 (see *p*-values in Additional file 14)

Up in *Gb* fiber			Up in *Gh* fiber		
GO ID	GO Name	FDR	GO ID	GO Name	FDR
GO:0042254	ribosome biogenesis	1.15E-09	GO:0015996	chlorophyll catabolic process	3.92E-06
GO:0042026	protein refolding	3.28E-05	GO:0010150	leaf senescence	3.92E-06
GO:0010143	cutin biosynthetic process	2.11E-04	GO:0019745	pentacyclic triterpenoid biosynthetic process	3.92E-06
GO:0006730	one-carbon metabolic process	2.01E-03	GO:0006090	pyruvate metabolic process	1.02E-05
GO:0006821	chloride transport	2.81E-03	GO:0009414	response to water deprivation	2.89E-05
GO:0006448	regulation of translational elongation	9.92E-03	GO:0006013	mannose metabolic process	1.23E-04
GO:0006536	glutamate metabolic process	1.00E-02	GO:0006094	gluconeogenesis	1.78E-04
GO:0009637	response to blue light	1.60E-02	GO:0006096	glycolysis	2.07E-04
GO:0015808	L-alanine transport	1.63E-02	GO:0006000	fructose metabolic process	3.34E-04
GO:0050732	negative regulation of peptidyl-tyrosine phosphorylation	1.63E-02	GO:0009651	response to salt stress	4.62E-04
GO:0051085	chaperone mediated protein folding requiring cofactor	1.63E-02	GO:0010193	response to ozone	1.11E-03
GO:0048640	negative regulation of developmental growth	1.63E-02	GO:0016556	mRNA modification	1.27E-03
GO:0072521	purine-containing compound metabolic process	2.76E-02	GO:0009965	leaf morphogenesis	1.34E-03
GO:0006639	acylglycerol metabolic process	2.76E-02	GO:0010363	regulation of plant-type hypersensitive response	1.97E-03
GO:0051302	regulation of cell division	2.76E-02	GO:0052542	defense response by callose deposition	2.47E-03
GO:0006909	phagocytosis	2.76E-02	GO:0030048	actin filament-based movement	2.47E-03
GO:0046653	tetrahydrofolate metabolic process	2.76E-02	GO:0009834	secondary cell wall biogenesis	2.47E-03
GO:0006123	mitochondrial electron transport, cytochrome c to oxygen	2.76E-02	GO:0019643	reductive tricarboxylic acid cycle	3.29E-03
GO:0006497	protein lipidation	4.45E-02	GO:0010167	response to nitrate	4.00E-03
GO:0009220	pyrimidine ribonucleotide biosynthetic process	4.50E-02	GO:0000165	MAPK cascade	5.62E-03
GO:0015865	purine nucleotide transport	4.50E-02	GO:0045492	xylan biosynthetic process	6.01E-03
GO:0015721	bile acid and bile salt transport	4.50E-02	GO:0010413	glucuronoxylan metabolic process	6.01E-03
GO:0015701	bicarbonate transport	4.50E-02	GO:0050832	defense response to fungus	6.46E-03
GO:0009116	nucleoside metabolic process	4.59E-02	GO:0051567	histone H3-K9 methylation	6.99E-03
GO:0008033	tRNA processing	4.74E-02	GO:0071805	potassium ion transmembrane transport	6.99E-03
			GO:0043068	positive regulation of programmed cell death	6.99E-03
			GO:0010200	response to chitin	8.09E-03
			GO:0000910	cytokinesis	9.02E-03
			GO:0019344	cysteine biosynthetic process	9.46E-03
			GO:0007030	Golgi organization	1.03E-02

### Overview of changes in the metabolome of 10 to 28 DPA cotton fiber

The metabolites are definitive indicators of cellular activity, whereas transcripts may not be translated. Therefore, we compared metabolite concentrations across the time-course of fiber development within one genotype and at the same DPA across genotypes (Fig. 1). Of 206 total metabolites, 194 or 201 were present in 10 DPA *Gb* or *Gh* fiber, with similar numbers detected at all other DPA. The 20 occurrences of the highest concentration metabolites in *Gb* or *Gh* fiber are shown in Additional file 15, and these will be discussed below in their biological context.

In *Gh* or *Gb* fiber, 44 or 45 metabolites did not show a significant change between 10 to 28 DPA. Of these, 18 were unchanged in both genotypes, including several sugars and sugar derivatives (such as xylitol, sucrose, mannose-6-phosphate, and UDP-glucose) that relate to the importance of turgor pressure and polysaccharide synthesis for cotton fiber morphogenesis.

A total of 161 or 162 metabolites (approximately 78 % of total metabolites) had significantly different concentrations across DPA in *Gb* or *Gh* fiber (p ≤ 0.05); (Additional file 3). The greatest metabolic change occurred between 21 to 28 DPA in both genotypes, in contrast to the decrease in transcriptional complexity in the same interval. Compared to 21 DPA, 99 or 95 different metabolites were more concentrated in *Gb* or *Gh* fiber at 28 DPA (or 73 or 62 metabolites, excluding dipeptides) (Fig. 1). Metabolites with higher concentration at 28 DPA accounted for most of the >10-fold changes across DPA (Additional file 15). In *Gb* fiber, 21 different metabolites showed 10 to 126-fold concentration increases as fiber development progressed, and 20 of 21 cases reflected higher concentrations at 28 DPA as compared to 10 or 21 DPA. Similarly, *Gh* fiber had 15 different metabolites with 10 to 72-fold concentration increases across DPA, and 13 of 15 cases reflected the same developmental contrasts. Nine metabolites with ≥10-fold concentration differences across DPA were held in common between *Gh* and *Gb* fiber: adenosine-, cytosine-, guanosine-, or uridine-2’,3’-cyclic monophosphate; linoleamide; methyl-beta-glucopyranoside; fucosterol; phytosphingosine; and raffinose, and these are discussed in the context of metabolite clusters below.

**Fig. 3:**
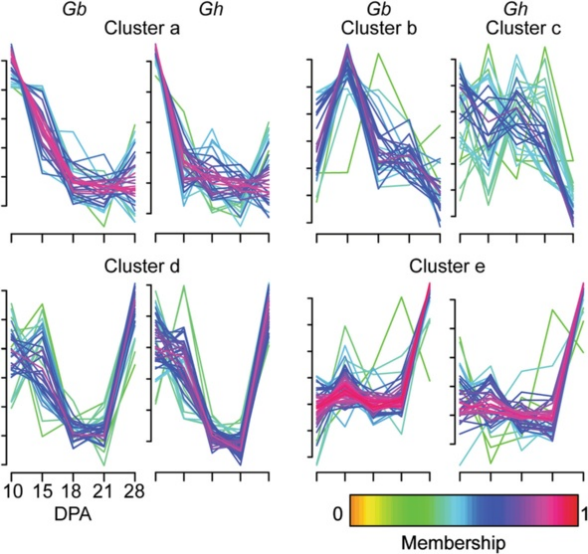
Four clusters of *Gb* and *Gh* fiber metabolites included three that were similar between genotypes. Metabolites with changes in concentration during fiber development (*p* ≤ 0.05) were clustered. The x-axis is as defined for Fig. 2. The y-axis is the scaled imputed mean value of metabolite concentration standardized to mean = 0 and standard deviation = 1. Color coding is as described in Fig. 2

Fuzzy C-means clustering revealed four major patterns of change in metabolite concentrations, with only three clusters being similar between genotypes (Fig. 3). Below we interpret the metabolite clusters by reference to metabolites with >10-fold concentration changes across DPA in both *Gh* and *Gb* fiber. Cluster ‘a-metabolites’ have higher concentration at 10 to 15 DPA with similar lower concentrations observed in both genotypes at 18 to 28 DPA. This cluster contains fucosterol (3β-Hydroxy-5,24(28)-stigmastadiene, Additional file 16). Phytosterols help to structure plasma membrane lipid domains that contain sphingolipids, which are derived from precursor phytosphingosine (4-D-Hydroxysphinganine) [50]. Phytosphingosine was in the ‘a’ or ‘c’ metabolite clusters in *Gb* or *Gh* fiber, but, like fucosterol, it occurred in high concentration at 10 DPA in both genotypes. Possibly, fucosterol and phytosphingosine are related to the organization of the plasma membrane to support high-rate elongation. The overall dissimilar Cluster ‘b-metabolites’ (*Gb*) and Cluster ‘c-metabolites’ (*Gh*) each contained linoleamide, with high concentration at 15 DPA in both genotypes. Linoleamide is derived from linoleic (18:2) fatty acid and modulates calcium levels in animal cells [51]. These two clusters (with 30 or 35 metabolites in *Gb* or *Gh* fiber) share the feature of lower metabolite concentrations at 28 DPA. About half of the molecules are held in common, including many fatty acids that could relate to building plasma membranes to support elongation. However one-third of the metabolites in *Gb* Cluster ‘b-metabolites’ have peak concentrations at 15 DPA, with potential functional consequences still to be determined. Cluster ‘d-metabolites’ are low at 18 to 21 DPA during transitional cell wall remodeling, but none showed >10-fold change across DPA in both genotypes. The biological context of this cluster will be discussed below as part of defining the transition stage. Cluster ‘e-metabolites’ have highest concentrations at 28 DPA in both genotypes and include: methyl-beta-glucopyranoside, raffinose, and the four nucleotide-2’,3’-cyclic monophosphates. The synthesis of methyl-beta-glucopyranoside may serve to detoxify methanol arising from pectin methylesterase activity during SCW synthesis [25, 52–54]. Raffinose is a sucrose-derived oligosaccharide that can act as an antioxidant to protect against oxidative stress, as well as exerting other protective effects [55]; see further discussion on ROS management below. The nucleotide-2’,3’-cyclic monophosphates are intermediate products of RNase activity that may signify increased nuclease activity in the initial stages of fiber cell death [56].

### Differences in the metabolome between *Gh* and *Gb* fibers

The difference in metabolite concentrations between *Gh* and *Gb* fiber was also determined at each DPA analyzed. *Gh* fiber had 105 metabolites with higher concentration than in *Gb* fiber, whereas 70 metabolites were more concentrated in *Gb* fiber (Additional file 3). Both genotypes differentially accumulated carbohydrates and amino acids, including ones associated with the metabolism of glutathione and the cellular control of redox homeostasis [46]. *Gh* fiber had 32 more dipeptides than *Gb* (see further discussion below). *Gb* fibers had higher levels of lipids, including three additional oxylipins that derive from the oxidation of linoleic or linolenic acid and participate in plant stress signaling [42, 57, 58]. *Gb* also had higher levels of six flavonoids (leucocyanidin, naringenin-7-O-glucoside, catechin, epicatechin, gallocatechin and epigallocatechin), whereas *Gh* fiber had higher levels of three other flavonoids (dihydromyricetin, dihydrokaempferol, and kaempferol 3-O-betaglucoside). These flavonoids may be acting as antioxidants or playing a role in modulating auxin signaling [59].

**Fig. 4:**
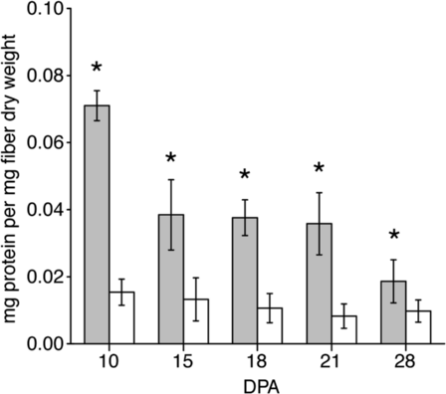
Total protein content in 10 to 28 DPA *Gh* and *Gb* fiber. Total protein was determined by Bradford assay and normalized to the dry weight of *Gb* (grey bars) and *Gh* (white bars) fiber. Mean values ± SD derive from 5 biological replicates at each DPA. Asterisks indicate a significant difference (p ≤ 0.017) between genotypes at each DPA as determined by t-test

On each DPA, *Gh* fiber had 53–78 metabolites with higher concentration than *Gb* fiber, whereas *Gb* fiber had 21–47 higher concentration metabolites as compared to *Gh* fiber (Fig. 1). There were 23–33 dipeptides that were more highly concentrated on each DPA in *Gh* fiber, with only glycyltryptophan having higher concentration in *Gb* fiber (Additional file 3). The list of metabolites with greatest concentration difference (≥8-fold) in *Gh* as compared to *Gb* fiber showed 12 of 25 cases occurring at 10 DPA, with the list dominated by dipeptides (Table 4). Here, the ≥8-fold threshold was chosen to focus the discussion, and one metabolite is sometimes listed on multiple DPA. More dipeptides in *Gh* fiber contrasted with higher total protein content in *Gb* fibers throughout fiber development (Fig. 4). Overall the data support more active protein turnover in *Gh* fiber. The list of metabolites with ≥8-fold difference in *Gb* as compared to *Gh* fiber was dominated (13 of 20 cases) by antioxidants (including flavonoids, glutathione and ascorbate) at diverse DPA, although 7 of 20 cases occur at 28 DPA (Table 4). The section on ROS management contains additional related discussion.

**Table 4:** Occurrences of ≥ 8-fold metabolite concentration differences between *Gb* and *Gh* fiber. Concentration differences were calculated from data in Additional file 3, which contains the *p*-values. FDR q-values were determined as in Table 1

	Higher concentration in *Gb* fiber					Higher concentration in *Gh* fiber		
DPA^a^	Biochemical	Fold- change	FDR		DPA^a^	Biochemical	Fold-change	FDR
28 DPA	ascorbate	138.27	0.00		10 DPA	serylleucine	38.82	0.00
15 DPA	leucocyanidin	79.89	0.00		10 DPA	dihydrokaempferol	17.02	0.00
21 DPA	ascorbate	50.36	0.04		10 DPA	N-acetylmethionine	16.11	0.00
18 DPA	ascorbate	46.12	0.02		10 DPA	adenosine	12.53	0.00
15 DPA	catechin	30.18	0.00		10 DPA	valylisoleucine	11.81	0.00
10 DPA	gallocatechin	23.37	0.00		10 DPA	histidylleucine	11.53	0.00
10 DPA	glutamate	23.27	0.00		10 DPA	leucylglutamate	11.44	0.00
28 DPA	adenosine 5’-diphosphate (ADP)	22.51	0.00		10 DPA	lysylleucine	11.12	0.00
28 DPA	leucocyanidin	17.02	0.00		10 DPA	threonylleucine	11.10	0.00
10 DPA	leucocyanidin	13.84	0.01		18 DPA	phenethylamine^b^	11.07	0.00
28 DPA	catechin	12.80	0.00		21 DPA	phenethylamine^b^	10.86	0.00
18 DPA	glutathione, reduced (GSH)	12.61	0.00		10 DPA	alanylleucine	10.59	0.00
28 DPA	gallocatechin	12.49	0.00		10 DPA	sucrose	9.27	0.00
15 DPA	gallocatechin	11.40	0.00		10 DPA	serylphenyalanine	9.25	0.00
15 DPA	epicatechin	11.26	0.00		10 DPA	alanylvaline	9.23	0.00
28 DPA	stachyose	10.83	0.00		10 DPA	alanylphenylalanine	8.86	0.00
10 DPA	caffeate	9.31	0.00		15 DPA	histidylleucine	8.85	0.00
21 DPA	glutamate	9.07	0.03		18 DPA	thymidine	8.69	0.00
18 DPA	glutamate	8.83	0.00		10 DPA	glycylleucine	8.61	0.00
28 DPA	glucarate	8.51	0.00		10 DPA	valylleucine	8.60	0.00
					15 DPA	alanylisoleucine	8.38	0.00
					15 DPA	alanylvaline	8.20	0.00
					18 DPA	adenosine	8.19	0.00
					10 DPA	inosine	8.15	0.00
					10 DPA	aspartylleucine	8.00	0.00

^a^DPA at which the high fold change relative to the other fiber genotype was observed; ^b^isobar with 1-phenylethanamine

### Transitional cell wall remodeling is a distinct developmental stage

**Fig. 5:**
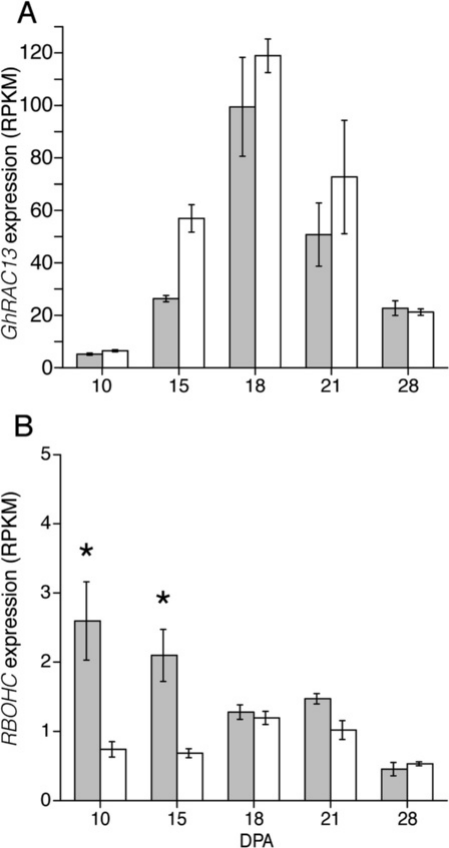
Expression of *GhRAC13* peaks during transitional cell wall remodeling. (**a**) The level of expression of *GhRAC13* (GenBank: S79308.1) is shown for *Gb* (grey bars) and *Gh* (white bars) fiber at 10 to 28 DPA, as derived from the RNA-Seq data. In both fibers, the similar timing of upregulated expression corresponds to the similar timing of the transition stage. (**b**) Expression of an RBOHC homolog (related to Gorai.001G053300.1) that may contribute to the oxidative burst by producing superoxide. Asterisks indicate a significant difference in the cross-genotype comparison (*p* ≤ 1E-4). Error bars are standard deviation

The data show that transitional cell wall remodeling is a distinct phase of fiber development that remains relatively stable at both the transcriptional and biochemical level for four days (between 18 to 21 DPA) under these experimental conditions. Only 0 or 2 transcripts and 6 or 8 metabolites changed concentrations between 18 and 21 DPA in *Gb* or *Gh* fiber, in contrast to substantial differences between 15 and 18 DPA and even greater differences between 21 and 28 DPA (Fig. 1). The expression pattern of the gene encoding a small GTPase-family protein (*GhRAC13*) [GenBank S79308.1], which is homologous to Gorai.011G031400.2 and AtRAC2 (At5g45970), confirmed the consistency of these results with known aspects of the transition stage in cotton fiber [60]. In the RNA-Seq data, *GhRAC13* or its ortholog in *Gb* fiber were upregulated between 15 to 21 DPA (Fig. 5a). They were also the most highly expressed RAC homologs at 18 DPA (Additional file 17), consistent with the prior data on *Gh* fiber [34].

The GhRAC13 protein is thought to regulate the activity of NADPH oxidase, a producer of the H_2_O_2_ signal required to initiate SCW synthesis [60, 61]. Plant Ras/Rho GTPases activate NADPH oxidases, which generate superoxide by transferring electrons from NADPH inside the cell across the membrane and coupling them to molecular oxygen to produce superoxide anion. The superoxide anion is then converted to H_2_O_2_ either spontaneously or by superoxide dismutase [29]. Only one NADPH oxidase transcript had RPKM > 2 (related to Gorai.001G053300 .1). It was more highly expressed in *Gb* at 10 and 15 DPA, but similarly expressed in both genotypes at 18–28 DPA including lower expression after the transition stage (Fig. 5b). This transcript is homologous to RBOHC/RHD2 (E-value = 0, Additional file 17) with a role in Arabidopsis root hair elongation [62]. The activity of NADPH oxidase is influenced by post-translational modifications and calcium [63], which may help to explain its somewhat divergent expression pattern between the genotypes as contrasted with the highly similar *RAC13* expression pattern. The expression of other transcripts potentially involved in the oxidative burst was generally similar between genotypes, except for one additional Arabidopsis *RAC2* homolog that was expressed in *Gh* but not *Gb* fiber (related to Gorai.010G242900.1) (Additional file 18).

Curiously Cluster ‘d-transcripts’, which were upregulated during transitional cell wall remodeling, were mirrored by Cluster ‘d-metabolites’ with lower concentrations at 18 and 21 DPA. As reported before for *Gh* fiber [24], the concentrations of glucose and fructose decreased beginning at 18 DPA with a continued decline until 28 DPA in both genotypes (Additional file 3). There were 33 or 46 metabolites in Cluster ‘d-metabolites’ for *Gb* or *Gh* fiber (30 or 25, excluding dipeptides). Nine of these metabolites were held in common between the two genotypes and help to illustrate special features of the transition stage in both *Gh* and *Gb* fiber (Additional file 16). In addition to one amino acid (valine) and a related dipeptide (valylvaline), the seven other common metabolites with lower concentrations were adenine, cytidine, dihydromyricetin, guanosine, histidine, tryptophan, and uridine. Adenine is used to synthesize ATP, NAD, and FAD during respiration, which occurs at a lower rate during transitional cell wall remodeling in *Gh* fiber [24]. Similarly, histidine is a precursor of alpha-keto-glutarate in the TCA cycle, which would be required at lower levels when the respiration rate is reduced. Adenine, cytidine, guanosine, and uridine are precursors of RNA, and their lower concentrations may indicate reduced RNA synthesis during a relatively stable developmental phase with almost no transcriptional change in either fiber genotype. Tryptophan is an amino acid used in the synthesis of auxin and serotonin, which may inhibit auxin activity in plants [64]. Similarly, dihydromyricetin is a flavonoid that mediates ethanol effects on the brain in interaction with γ-aminobutyric acid (GABA) and glutamate [65], both of which are present in Cluster ‘a-metabolites’ with high concentration during elongation of *Gh* and *Gb* cotton fiber. In plants, glutamate is a newly emerging signaling molecule [66] as well as a precursor to ROS scavenging glutathione (see below), whereas GABA modulates the activity of Ca^++^ channels [67]. Therefore, the lower concentrations of tryptophan and dihydromyricetin at 18 to 21 DPA likely signify shifts in intracellular signaling at this distinctive developmental stage.

### *Gh* and *Gb* fiber show similar expression profiles for cellulose synthase (*CESA*), and cellulose-synthase-like (*CSL*) genes

Glycosyltransferases within the cellulose synthase superfamily, including cellulose synthases (*CESA*s) and cellulose-synthase-like transcripts (*CSL*s), are required to produce para-crystalline cellulose fibrils and the polysaccharides in the cell wall matrix [17, 18]. The expression pattern of this gene family is often similar between *Gh* and *Gb* fiber, serving to highlight the logical correlation of gene expression with the similar early fiber elongation rates and timing of the onset of SCW formation in the two genotypes.

*CESA genes: Arabidopsis thaliana* provides a reference point for groups of *CESA* genes in seed plants [17]. The ten Arabidopsis *CESA* genes are grouped into six clades typified by: *AtCESA1* (and including *AtCESA10*)*; AtCESA3; AtCESA4; AtCESA6* (and including *AtCESA2,5,9*); *AtCESA7;* and *AtCESA8*. The synthesis of expanding primary walls (as in growing roots and hypocotyls) requires the activity of AtCESA1, 3, and 6, whereas SCW synthesis (as in xylem tracheary elements and interfascicular fibers) requires the activity of AtCESA4, 7, and 8. For both primary and secondary wall synthesis, a distinct heteromeric cellulose synthesis complex contains at least 18 total CESAs [68]. The fifteen *CESA* loci in the *Gr* genome [47], as compared to 10 *CESA* loci in Arabidopsis, is generally consistent with the comparative gene richness in these two species [69].

**Fig. 6:**
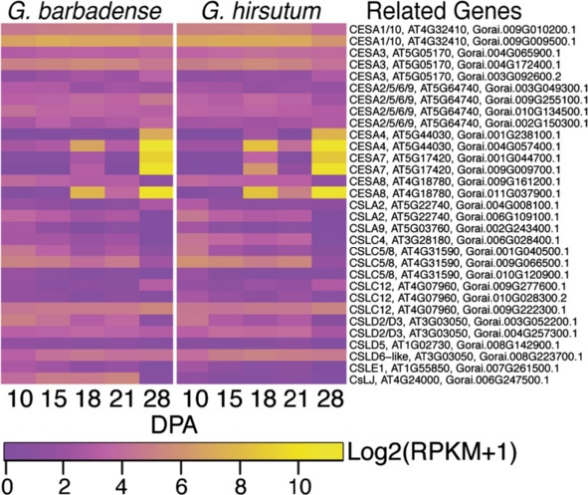
Expression patterns of CESA and CSL genes in *Gb* and *Gh* fibers. Heat map colors correspond to the LOG2 transformed RPKM value plus one for each transcript analyzed. Higher and lower expression levels are shown in yellow or purple, respectively. The data from cotton transcripts are referenced to ‘Related genes’ including the gene name and locus identifier for the most homologous Arabidopsis gene and the transcript identifier for the most homologous *Gr* gene

Both *Gh* and *Gb* fiber express genes mapping to each of the 15 *Gr CESA* loci (Additional file 17), with remarkable similarity in pattern and magnitude of expression of each isoform. There were 10–15 *CESA* loci expressed on the days analyzed here (Fig. 6). As expected, loci homologous to *AtCESA1, 3,* and *6* were expressed during cotton fiber elongation, although the expression of transcripts in the *AtCESA2, 5, 6, 9* clade was low overall. Expression of these ‘primary wall CESAs’ persists through 28 DPA even in *Gh* fiber where elongation had stopped five days earlier. Transcripts homologous to one of the *AtCESA8-like* loci (and Gorai.009G161200.1) are downregulated at 28 DPA in both genotypes, suggesting that an AtCESA8-like CESA may have adopted a novel role in early elongating cotton fiber (Additional file 17).

The homologs of *AtCESA4, 7,* and *8* were upregulated at 18 and 28 DPA during early and late cell wall thickening (Fig. 6). Interestingly, these show less expression at 21 DPA, consistent with the relatively static stage of transitional cell wall remodeling and winding layer synthesis between 18 and 21 DPA. All 15 *CESA* loci were expressed at 18 DPA during transitional cell wall remodeling, showing the potential for cellulose synthesis complexes of diverse types to be formed within cotton fiber if all of the transcripts are translated. Expression of many *CESA* loci in multiple clades continued at 28 DPA, even in *Gh* fiber that was no longer elongating.

*CSL genes:* The CESA superfamily includes 9 cellulose-synthase-like (CSL) families in addition to CESAs, inclusive of clades CSLA to CSLH and CSLJ. The CSLs have motifs indicative of ß-glycosyltransferase activity, and some have been proven to participate in synthesis of the hemi-cellulosic cell wall matrix polysaccharides including xyloglucan and mannan [18, 70]. The *Gr* genome has at least 35 *CSL* loci within the CSLA to CSLE, CLSG, and CSLJ families, compared to 31 *CSLs* in Arabidopsis [47]. Both *Gh* and *Gb* fiber express 16 *CSL* genes within four families (CSLA, CSLC, CSLD, CSLE), although the expression of the only CSLE homolog is weak (Additional file 17). At least one gene in the CSLA, CSLC, and CSLD, families is expressed relatively strongly from 10 to 21 DPA in elongating fiber of both genotypes. In addition, representatives of the CSLC and CSLD families are expressed strongly at 28 DPA when only *Gb* fiber is continuing to elongate. *Gb* fiber at 10 to 21 DPA expresses a homolog of a fifth CSL family, CSLJ, which exists in many angiosperms (but not Arabidopsis) and has unknown function [70] (Fig. 6).

Arabidopsis CSLC4 (with one cotton fiber homolog) supports the synthesis of the ß-1,4-glucan backbone of xyloglucan [71], which is found within the primary wall of *Gh* and *Gb* fiber [10]. Cotton fiber shows relatively weak expression of a homolog of *AtCSLA2* and even lower expression of an *AtCSLA9* homolog: both of the encoded proteins have ß-1,4-mannan and/or glucomannan synthase activity *in vitro* [72]. Mannan has been detected in extracts of tightly bound cell wall matrix polymers of 6 to 30 DPA *Gh* fiber and mature *Gh* and *Gb* fibers that are still surrounded by a primary wall [25, 73]. Tentatively, it may contribute positively to the ‘elongation’ parameter, or the extent of fiber stretching before breaking [73]. Members of the CSLD family can support the synthesis of cellulose, cellulose-like polymers, and/or mannan. One cotton gene homologous to *AtCSLD2/3* (two closely related isoforms in Arabidopsis) has strongest expression at 10 DPA in both genotypes, and another is expressed at a low level throughout 10 to 28 DPA. Proteins in the CSL2/3 clade are required for normal root hair growth in diverse plant species [74, 75], and their roles in cotton fiber can be explored in further research.

### Cotton fiber modifies an ancient gene regulatory network to repress lignification

**Fig. 7:**
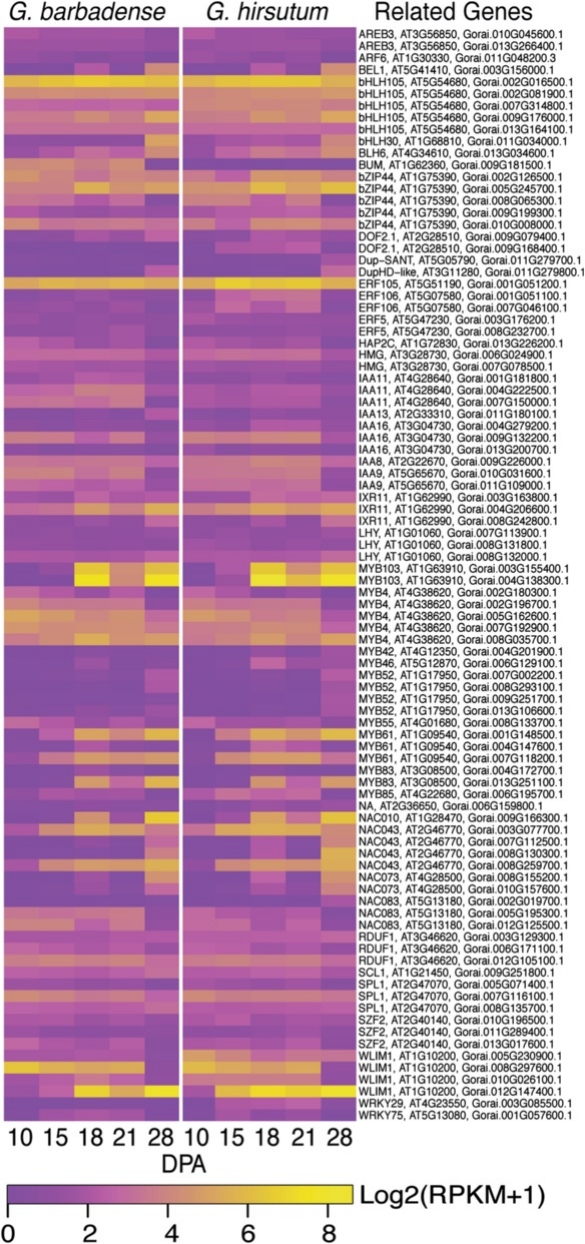
Expression patterns of genes encoding secondary wall related transcription factors in *Gb* and *Gh* fibers. The heat map parameters are explained in the legend of Fig. 6

The genetic program for cotton fiber SCW synthesis resembles the ancient hierarchical gene network that regulates the differentiation of sclerenchyma cells [6, 15, 76, 77]. However, unlike those cell types in the plant body, cotton fiber represses hemicellulose, protein, and lignin biosynthesis during SCW synthesis so that cellulose becomes predominant. Our data show that cotton fiber expresses 92 primary loci that are homologous to 45 transcriptional regulators of Arabidopsis sclerenchyma cell differentiation [19, 20] (Additional file 17). Of these, 39 cotton homologs have fold-change ≥2 between 10 to 28 DPA in either *Gh* or *Gb* fiber, and the expression patterns are highly similar in both genotypes (Fig. 7).

Our data showed that cotton fiber expresses genes that are highly homologous to Arabidopsis SCW transcriptional regulators named *NST1*, *SND2*, *SND*, *MYB4*, *MYB42*, *MYB46*, *MYB52*, *MYB55*, *MYB61*, *MYB83*, *MYB85*, *MYB103*, *KNAT7*, *VNI2*, myb-like (At3g11280), *BLH6*, and *RDUF1* (Additional file 17). We will draw analogies below to gene/protein functions in Arabidopsis sclerenchyma cells [20, 22, 77], although sequence changes in *Gossypium* may also affect the precise regulatory roles in cotton. The transcriptional network controlling sclerenchyma SCW synthesis is described in hierarchical tiers with higher levels having broader control of SCW differentiation processes [20]. Cotton fiber expresses only one of five ‘Tier 3’ master regulators of SCW synthesis: homologues of *NST1* (*NAC043*) that is active in Arabidopsis stem fibers (but not vessels). In both cotton genotypes, two of four *NST1* homologues were upregulated at 15 DPA, just before transitional cell wall remodeling, and remained highly expressed through 28 DPA (Fig. 7). The other two loci were upregulated more strongly in 28 DPA *Gh* fiber than in *Gb*, pointing to potential isoform-specific effects on the extent of SCW thickening. Other genes, such as a homolog of BLH6 that appears to activate SCW thickening in Arabidopsis stem fibers, may also help to regulate cotton fiber SCW synthesis.

Cotton fiber expresses five of seven ‘Tier 2’ transcription factor targets of NST1, each of which is independently capable of inducing SCW synthesis in Arabidopsis (Fig. 7). In cotton fiber, *MYB61* was upregulated at 15 DPA like *NST1*, whereas the expression of *SND3 (NAC010), MYB83*, and *MYB103* were strongly upregulated by 18 DPA. The expression of a single locus homologous to *MYB46*, which is redundant in Arabidopsis with *MYB83*, was weakly and transiently upregulated at 18 DPA. At least some homologs of *SND3*, *MYB83*, and *MYB103* showed a high-low-high expression pattern at 18-21-28 DPA, which correlates with the relatively stable phase of transitional cell wall remodeling at 18 to 21 DPA followed by the distinct phase of mainly cellulose synthesis. These transcription factors may interact with different partners to modulate the synthesis of different layers as the cotton fiber thickens, i.e. the thin transitional “winding” layer followed by the thick SCW composed primarily of cellulose.

In correlation with dominance of cellulose synthesis during SCW thickening, the cotton fiber genotypes analyzed here expressed only five homologs of the 19 ‘Tier 1’ transcription factors (Fig. 7). In Arabidopsis, the ‘Tier 1’ transcription factors control particular SCW processes such as cellulose, hemicellulose, and lignin synthesis [20]. The expression of three *KNAT7 (IXR11)* homologs at variable levels over time in cotton fiber is consistent with the modulation of the amount of secondary wall cellulose, which is important because overly thick walls prevent cotton fibers from drying with the ‘kidney bean’ shape required for yarn spinning [78]. In other plants, *KNAT7* can activate or repress SCW synthesis in different cell types, depending on its protein partners. A *Gh* ortholog of *AtKNAT7* (KC200250, related to Gorai.004G206600.1) can modulate the SCW thickness of interfasicular fibers in transgenic Arabidopsis, although the mechanism behind the effect was unclear [79], and this gene was upregulated between 18 to 28 DPA in our data. Other ‘Tier 1’ regulators expressed in cotton fiber include: 2 loci homologous to *SND2* (*NAC073*), both with upregulated expression only at 18 and 28 DPA; and 2–4 loci of *MYB52* with weak expression at 28 DPA. The roles of SND2 and MYB52 in sclerenchyma cells are not completely clear, although MYB52 may repress lignification or the whole SCW differentiation program [19, 20]. In addition to recognized ‘Tier 1’ regulators, cotton fiber also shows strongly upregulated expression between 15 to 28 DPA of one of four loci homologous to Arabidopsis *LIM1* (*WLIM1*; related to Gorai.012G147400). Over-expression in cotton of another *LIM1* homolog [GenBank: JX648310], which is related to Gorai.010G026100.1, resulted in ~20 % thinner fibers with 1.4 % lignin/lignin-like-phenolics compared to 1 % in wild type [80]. Therefore, this gene family may help to control the balance between cellulose and lignin content in cotton fibers.

Corresponding to little or no lignin in the fiber of advanced commercial cotton cultivars [81], the cotton homologs of nine lignin-regulating transcription factors (*MYB20, MYB58, MYB63, MYB69, MYB75, MYB79, ATHB18, BP*, At3g46080) [20] were not expressed at RPKM ≥ 2 (Additional file 17). In contrast, cotton fiber expresses 5 loci homologous to *MYB4*, which is a widely conserved repressor of lignification in angiosperms [22] (Fig. 7). One of the *MYB4* loci was strongly upregulated at 28 DPA during the synthesis of SCW with little or no lignin, perhaps indicating a specific role for this isoform in repressing SCW lignification in cotton fiber. In addition, cotton fiber expresses homologs of *MYB*-like At3g11280 and *RDUF1*, which repress lignification and/or the whole SCW differentiation program in Arabidopsis [19]. In cotton fiber, one locus homologous to At3g11280 was upregulated at 28 DPA, implying that the encoded protein may indeed repress lignification specifically. Conversely, the cotton gene encoding RDUF1-like protein was downregulated at 28 DPA, suggesting that it may act as repressor of SCW cellulose synthesis. Similarly, the transcription of three loci encoding homologs of *VNI2* (*NAC083*), a SCW repressor in Arabidopsis, shows a general pattern of downregulation at 28 DPA when cotton fiber is undergoing massive SCW thickening.

**Fig. 8:**
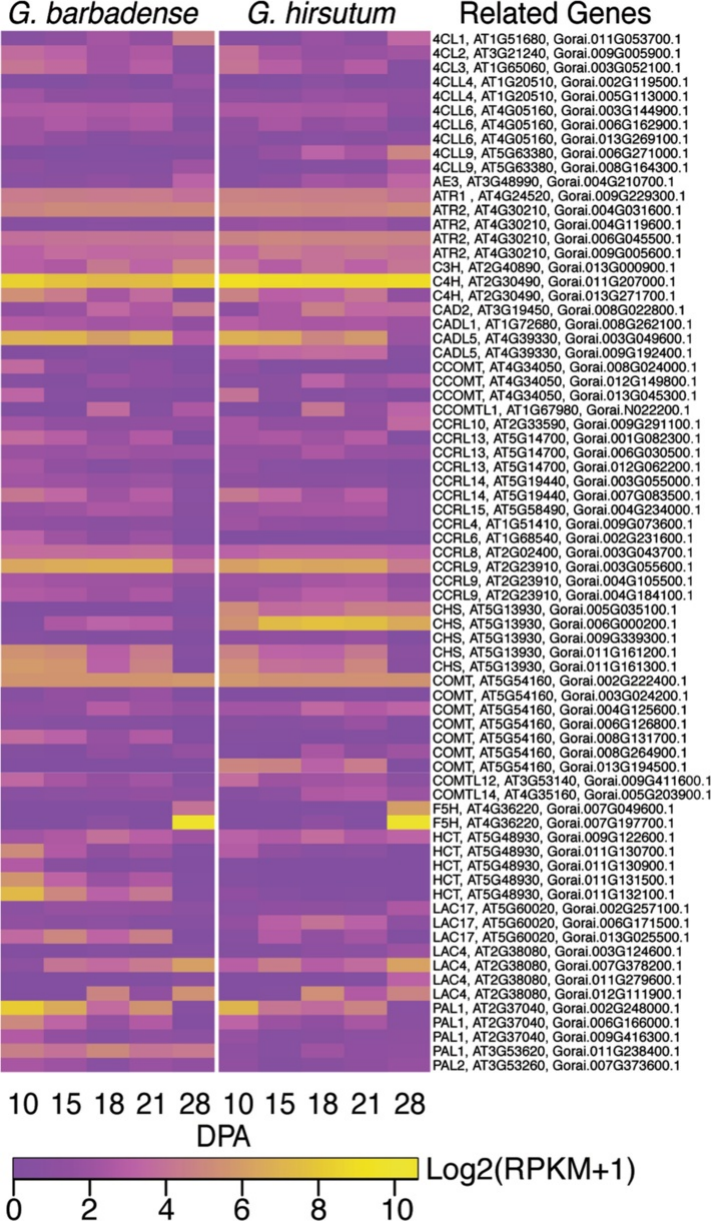
Expression patterns of genes related to lignin biosynthesis in *Gb* and *Gh* fibers. The heat map parameters are explained in the legend of Fig. 6

The results show that lignin synthesis in commercial cotton fiber is repressed at the transcriptional level. Consistently, no lignin monomers (p-coumaroyl-, coniferyl-, or sinapyl-alcohols) were found in the fiber metabolome, although monolignols were readily detected even in young tree shoots by similar methods [82] (Additional file 3). Nonetheless, 74 loci homologous to lignification-related structural genes [20, 21] (Additional file 17) were expressed in at least one cotton fiber sample, often at low levels. Transcripts encoding three enzymes needed to convert phenylalanine to p-coumaryl CoA were detected (phenylalanine ammonia lyase, PAL; C4-hydroxylase, C4H; and 4-coumaroyl-CoA-ligase, 4CL, or 4CL-like). However, these enzymes support the synthesis of both lignin monomers and flavanoids [83], which were abundant in the cotton fiber metabolome. The flavonoid branch of the phenylpropanoid pathway is modulated by chalcone synthase (CHS, with 3-5 loci expressed in cotton fiber). Alternatively hydroxycinnamoyl-CoA shikimate/quinate hydroxycinnamoyl transferase (HCT) controls flux toward lignin. *Gb* fiber had stronger expression of more *HCT* loci as compared to *Gh* fiber, but both genotypes had low expression of only one *HCT* locus at 28 DPA during SCW synthesis (Fig. 8). Therefore, flux toward lignin may be limited during cotton fiber SCW synthesis despite the (overall low) expression of down-stream genes on the lignin branch pathway. The expression patterns of lignin-related structural genes are displayed in Fig. 8 including homologues of: C3-hydroxylase (*C3H*); cinnamoyl-CoA reductase-like (*CCRL*); cinnamyl alcohol dehydrogenase (*CAD* or *CAD-like*); ferulate 5-hydroxylase (*F5H*); caffeoyl-CoA O-methyltransferase (*CCOMT* of *CCOMT*-like); and caffeic acid O-methyltransferase (*COMT* or *COMT*-like). These could be related to the presence of minor amounts of lignin-like phenolics in cotton fiber [28, 80, 81].

### Differences in ascorbate synthesis and recycling may enhance ROS management and elongation in *Gb* cotton fiber

Previous indications of the importance of ROS management in cotton fiber development and quality improvement were provided in the introduction. Here, we provide new insights into the genetic and biochemical basis of this effect. The fibers from *Gb* and *Gh* accumulate and manage antioxidants differently, especially at the transition stage and afterwards when increasing ROS levels help to regulate SCW synthesis while elongation in *Gb* fiber also continues. As detailed below, *Gb* fiber has greater capacity to manage ROS, which is consistent with its higher protein levels (as detected in Bradford assay) as contrasted with the higher levels of dipeptides (derived from protein degradation) in *Gh* fiber, as summarized previously.

**Fig. 9:**
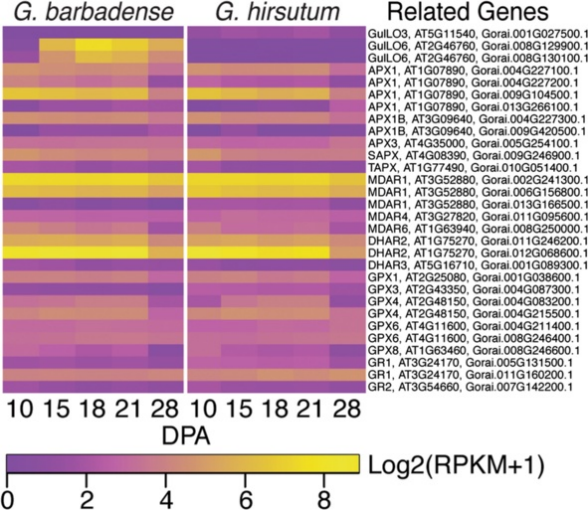
Expression patterns of genes related to ROS management via the glutathione-ascorbate cycle and the glutathione-perioxidase cycle in *Gb* and *Gh* fibers. The heat map parameters are explained in the legend of Fig. 6

**Fig. 10:**
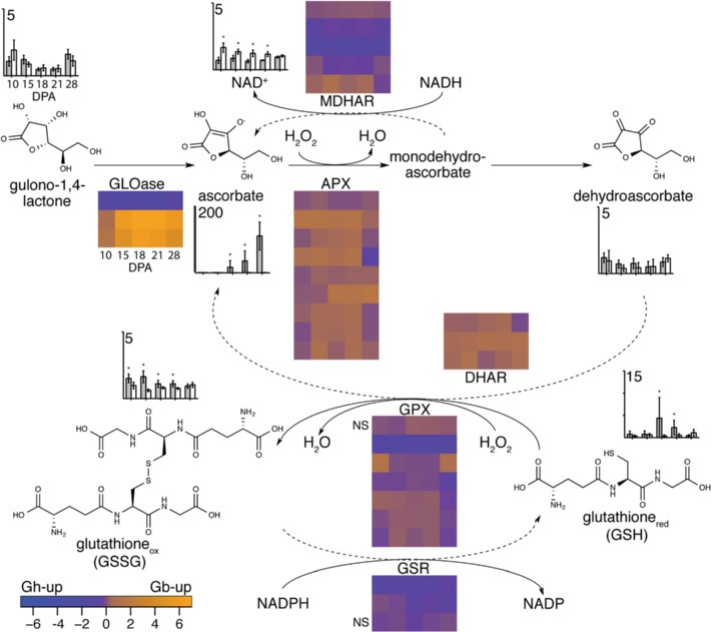
Ratio of transcripts and metabolite levels in the glutathione-ascorbate cycle and the glutathione peroxidase cycle in *Gb* and *Gh* fiber. The pathway is adapted from [93]. Metabolites: Graphs show the level of detected metabolites across DPA in *Gb* (grey bars) and *Gh* (white bars) fiber, with each X-axis tick representing 10, 15, 18, 21, or 28 DPA. The Y-axis shows the scaled imputed means for metabolite concentrations, with the maximum value shown numerically at the top. The detected metabolites are indicated by chemical structures, except for NAD^+^ that was omitted for clarity Oxidized or reduced glutathione are indicated with the subscripts ‘ox’ or ‘red’. Asterisks indicate a significant difference in the cross-genotype comparison (p ≤ 0.05). Ratios of transcripts: The heat maps show the ratio of expression of different loci in *Gb* vs *Gh* fiber, with each DPA represented on the horizontal. Each row represents a different gene, portrayed in the same order for each gene type as in Fig. 9. Enzyme names putatively corresponding to the transcripts are shown adjacent to reaction arrows. Forward or reverse reactions are indicated by solid or dashed lines. All of the transcripts shown had a significantly different expression level between *Gh* and *Gb* fiber on at least one DPA, except where NS is shown in two cases

The heat map of expression levels of genes related to ROS management via the glutathione-ascorbate cycle [84] is shown in Fig. 9, and the combined transcriptomic and metabolomics data are shown in Fig. 10. The relevant specialized cotton fiber metabolites that were detected in this study included gulono-1,4-lactone, ascorbate, dehydroascorbate, nicotinamide adenine dinucleotide (NAD+), and reduced and oxidized glutathione (GSH and GSSG). Relevant cotton fiber transcripts were homologous to genes encoding gulono-1,4-lactone oxidase (GLOase, or GulLO6), APX, monodehydroascorbate reductase (MDHAR or MDAR), DHAR, glutathione disulfide-reductase (GSR, or GR), and glutathione-dependent peroxidase (GPX) (Additional file 17). At neutral pH most ascorbate is in the form of ascorbate(H^−^), a reducing agent/anti-oxidant that readily donates a hydrogen atom/electron to H_2_O_2_, converting it to harmless H_2_O in a reaction modulated by APX. The (often short-lived) monodehydroascorbate product can donate a second electron to yield dehydroascorbate in a non-enzymatic process. Reduced ascorbate can be regenerated by: (a) MDHAR using monodehydroascorbate and NADH cofactor; or (b) DHAR using dehydroascorbate and reduced glutathione cofactor. In addition, reduced glutathione acts as an anti-oxidant through donating an electron to H_2_O_2_ or oxidized biomolecules, followed by formation of dimeric oxidized glutathione, as catalyzed by GPX enzyme activity. A low GSH:GSSG ratio has been used as a metabolic indicator of oxidative stress in plants [85], and GSR enzyme activity maintains the pool of GSH that is available for ROS scavenging and use as a co-factor during replenishment of the ascorbate pool via DHAR activity.

In contrast to the heat map in Fig. 9 that shows the levels of gene expression in both genotypes, the heat maps in Fig. 10 are ratios of gene expression between *Gb* and *Gh* fiber at each DPA. Only five genes have consistently higher expression in *Gh* fiber (dark purple rows), but these have very low expression in both fiber genotypes. These are homologs of *GLOase* (Gorai.001G027500), *MHDAR* (Gorai.013G166500 and Gorai.006G156800), *GPX* (Gorai.004G087300), and *GSR* (Gorai.005G131500). In following, we emphasize the many other genes with moderate to high expression levels that also have a higher *Gb*:*Gh* expression ratios.

#### Abundance and synthesis of antioxidants

The metabolomics analysis revealed a much higher level of ascorbate in *Gb* fiber at 18 to 28 DPA, with a 138-fold increase at 28 DPA as compared to *Gh* fiber. *Gb* fiber also expressed two *GLOase* genes at 365-5,385-fold higher level at 15 to 28 DPA (related to Gorai.008G129900 and Gorai.008G130100; both homologs of At2g46760, *AtGUlLO6*). This may be a general attribute of *Gb* accessions, a hypothesis that is supported by our analysis of published microarray data [7]; Additional file 19. In total, our data showed that only three *GLOase* homologs were expressed at RPKM ≥ 2 in cotton fiber. Interestingly, At2g46760 is in a co-expression network (http://atted.jp/cgi-bin/locus.cgi?loc=At2g46760) with the *VND7* transcription factor gene (At1g71930) that initiates SCW synthesis in water-conducting vessels [86] rather than supportive xylem fibers. The GLOase enzyme converts gulono-1,4-lactone into ascorbate, and gulono-1,4-lactone was detected at low similar levels in fibers of both genotypes. At all DPA tested both *Gh* and *Gb* fiber had similar levels of precursors of gulono-1,4-lactone biosynthesis, *myo*-inositol-1-P and *myo*-inositol. Collectively, the data support the possibility that a major difference in *GLOase* gene expression flows into increased GLOase protein and enzyme activity to produce more ascorbate in *Gb* fiber.

*Gb* fiber also had higher levels of one or both forms of glutathione at 10 to 21 DPA as compared to *Gh* fiber. Reduced glutathione was most concentrated at 18 DPA near the onset of cell wall thickening. Correspondingly, the level of glutamate, the precursor for glutathione, was also significantly higher in *Gb* fiber at 10, 18, and 28 DPA. The levels of transcripts homologous to glutathione synthetase or gamma-glutamylcysteine synthetase genes, which encode the enzymes needed for conversion of glutamate to glutathione, were not significantly different between genotypes.

#### Effects of ascorbate on cell elongation

In addition to acting as a general cellular reductant, ascorbate may also contribute to the production of highly reactive hydroxyl radicals in the apoplast. In the presence of copper, ascorbate reduces molecular oxygen to peroxide and Cu^++^ to Cu^+^ resulting in the formation of hydroxyl radicals through a Fenton reaction. The hydroxyl radicals can cleave cell wall polysaccharides such as xyloglucan and pectins, potentially increasing the fluidity of cell wall polymers and enabling cell expansion [87, 88]. This mechanism could operate during the prolonged elongation period of *Gb* fiber when ascorbate levels are high. Proving that ascorbate serves one or both roles during late elongation in *Gb* fiber will be aided by determining the sub-cellular localization of ascorbate and the extent of apoplastic hydroxyl radical production during and after the transition stage.

#### Use and replenishment of ROS scavengers

Both *Gh* and *Gb* fiber expressed numerous genes encoding APX, which mediates the use of ascorbate as a ROS scavenger. The levels of oxidized ascorbate, or dehydroascorbate, were low and similar in 10 to 28 DPA *Gh* and *Gb* fiber, which is consistent with H_2_O_2_ scavenging via ascorbate in both fiber types. Nine *APX* loci expressed in cotton fiber were homologous to five Arabidopsis *APX* genes, with most of them being more highly expressed in *Gb* fiber. The cellular ascorbate pool can potentially be diminished through the degradation of dehydroascorbate into 2,3-diketogulonate, or the reduced ascorbate pool can be replenished through DHAR and/or MDHAR enzyme activity. DHAR was expressed more strongly in *Gb* fiber, which may indicate its preferential use of the glutathione-dependent DHAR-mediated dehydroascorbate recycling pathway to promote fiber elongation. Consistent with this possibility, transgenic plant experiments showed that DHAR enhanced ascorbate content more than MDHAR [89], improved antioxidant capacity, reduced oxidation of lipids, and improved root growth [90].

**Fig. 11:**
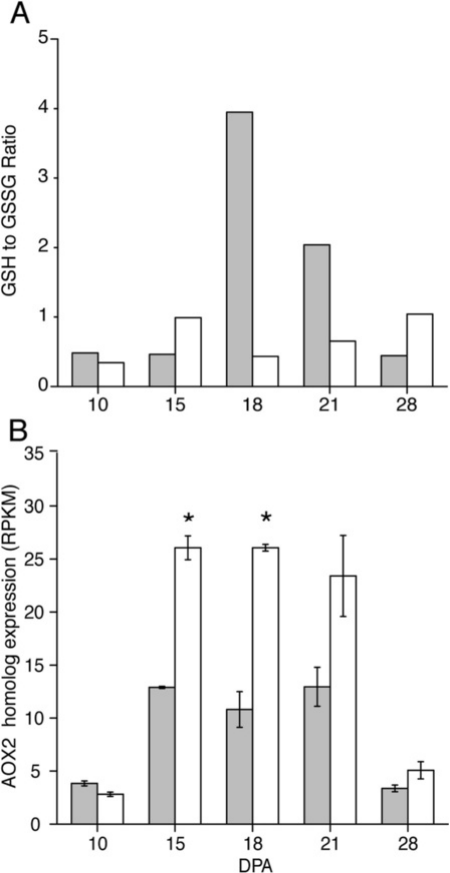
Metabolomic and transcriptomic evidence that transition-stage *Gh* fiber experiences more oxidative stress than *Gb* fiber. **a**) The ratio of reduced glutathione (GSH) to oxidized glutathione (GSSG) in *Gb* (grey bars) and *Gh* (white bars) fiber. The ratio is based on the scaled imputed mean for both metabolites. **b**) The expression level (RPKM) of a putative alternative oxidase (*AOX*) gene in *Gh* (white bars) and *Gb* (grey bars) fiber as derived from RNA-Seq data. The homologous protein in Arabidopsis (AOX2, At5G64210) attenuates ROS production during respiration [91], and the related *Gr* transcript is Gorai.005G220500.1. Asterisks indicate significant differences between genotypes at a given DPA. Error bars are standard deviation

Both genotypes showed generally similar *GPX* transcript levels, but two of three *GSR* transcripts were higher in *Gh* fiber. This difference may partly account for the 1.4–2.8-fold higher levels of oxidized glutathione in *Gb* from 10 to 21 DPA. Reduced glutathione was 4.5–12.6-fold higher in *Gb* at 18 and 21 DPA during transitional cell wall remodeling, and the GSH:GSSG ratio was higher in *Gb* fiber (Fig. 11a). Given that a high GSH:GSSG ratio indicates less oxidative stress in plant cells [85], this result is consistent with lower oxidative stress in *Gb* fiber as compared to *Gh* fiber. *Gb* fiber also had significantly lower levels of alternative oxidase transcript (related to Gorai.005G220500.1 and At5g64210/*AOX2*) at 15 and 18 DPA (with the same trend observed at 21 DPA) (Fig. 11b). *AOX* is induced during stress to attenuate ROS accumulation and energy production by bypassing the electron transport chain [91], and higher *AOX* levels are correlated with low fiber quality in the *im* cotton fiber mutant [92]. Higher oxidative stress in the fiber of *Gh* cv Deltapine 90 may be one explanation for its lower quality as compared to the fiber of *Gb* cv Phytogen 800.

#### Molecular and biochemical supports for increasing cotton fiber length

Our transcriptomic and metabolomic data support the prediction that it will be possible to generate longer fibers in *Gh* and other short fibered cotton (such as *G. arboreum* grown in Asia) through use of breeding or biotechnology strategies to enhance the levels of ascorbate in fiber, to use ascorbate for ROS scavenging via APX, and to recycle dehydroascorbate via DHAR. Over-expression of chloroplast-targeted *APX* provided preliminary indications of an approximately 10 % increase in *Gh* fiber length, while over-expression of *GR* had no effect compared to wild type [33]. Consistently, *Gb* fiber did not show any upregulation of *GR* gene expression in the RNA-Seq data. Our data reveal genes encoding particular isoforms of GLOase, MDHAR, APX, DHAR, and GPX that could be over-expressed to increase the quality of *Gh* fiber (indicated by orange colors assigned to higher *Gb/Gh* gene expression ratios in Fig. 10). In particular, we predict that over-expression of the *GLOase* genes that are uniquely and strongly expressed in *Gb* fiber (related to Gorai.008G129900 and Gorai.008G130100) will improve the length of *Gh* fiber. Higher expression of rat *GLOase* increased ascorbate levels in plants [93]. It may also be beneficial to increase the metabolic flux to gulono-1,4-lactone at the same time so that GLOase does not become substrate limited. Although we did not detect the alternative ascorbate precursor, L-Galactono-1,4-lactone, or any of its upstream metabolites [93] in 10 to 28 DPA cotton fiber, generating flux through this pathway might confer advantages in cotton fiber as well.

To identify other transcripts that might contribute to the sustained elongation of *Gb* fibers, we compared the intersection of three gene sets. The first gene set included *Gb* transcripts with ≥ 2-fold higher or similar expression level in the 28/21 DPA comparison, denoting continued expression during late-stage elongation in the fiber of *Gb* cv Phytogen 800. (Similar expression level was defined as < 2-fold change, or *Gb* 28/21 DPA ratio ≥ 0.5.) The second gene set included *Gh* transcripts with 21/28 DPA expression ratio of ≥ 2-fold, denoting a substantial decline in 28 DPA *Gh* fibers that were no longer elongating. The third gene set included transcripts that were expressed in either genotype at 10 DPA during early high-rate elongation, implying an assumption in this analysis that late-stage elongation in *Gb* fibers was mechanistically similar to early elongation. This assumption was supported by prior data showing that *Gb* fiber had a longer period of plasmodesmatal closure at the fiber foot as compared to *Gh* fiber. The resulting symplastic isolation is thought to increase the turgor pressure that contributes to fast elongation [94].

**Fig. 12:**
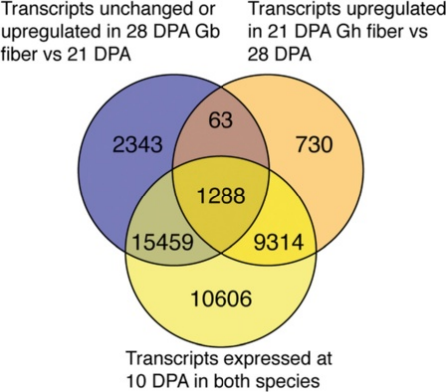
Venn diagram implicating transcripts that may contribute to the extended elongation period of *Gb* fibers. The three transcript sets compared were: (1) upregulated or unchanged from 21 to 28 DPA in *Gb* fibers; (2) downregulated from 21 to 28 DPA in *Gh* fibers; and (3) expressed in both genotypes at 10 DPA during early high-rate elongation. The 1,288 transcripts held in common may support the continued elongation of *Gb* fiber

These three data sets overlapped by 1,288 transcripts (Fig. 12). Of this subset, the transcripts most highly expressed in 28 DPA *Gb* fibers relative to 28 DPA *Gh* fibers include ones that encode proteins that can logically support continued elongation: a tonoplast intrinsic protein/aquaporin (AtTIP1;3-like); a pectin (pectate) lyase (At1G04680-like); and a UDP-glycosyltransferase (AtUGT73B4-like) (Additional file 20). For each of these highlighted cases (and others not discussed here), there is no other primary transcript mapping to the same Arabidopsis gene identifier in this Venn intersection set, supporting the possibility that the implicated functions and/or protein isoforms may support prolonged fiber elongation.

The aquaporin gene is homologous to water-transporting *AtTIP1;3* (in the Tonoplast Intrinsic Sub-family of aquaporins). This protein localizes to the vacuole and, together with AtTIP5;1, is required for normal Arabidopsis pollen tube growth [95, 96]. The aquaporin gene family in cotton is predicted to be important for the generation of turgor pressure to drive fiber elongation [97]. The pectin lyase is homologous to polygalacturonanase At3g48950, within a complex 67-member Arabidopsis gene family [98]. Pectin degradation may facilitate cell wall loosening and continuing fiber elongation [99]. For example, a pectin lyase with peak expression in 10 DPA *Gh* fiber positively regulates fiber elongation [100]. Arabidopsis UGT73B4 can glycosylate the flavanol anti-oxidant quercetin, which is expected to increase its solubility and stability [101]. A cotton UGT73B4-like protein may function similarly to support the role of flavonoids in ROS management. Related GO biological processes overrepresented in this dataset include: water transport, hydrogen peroxide catabolic process, and cell redox homeostasis (Additional file 21).

The metabolite data also revealed differences in 28 DPA *Gb* fiber that could support prolonged elongation. Of metabolites with ≥10-fold concentration difference across DPA, 11 of 29 were more concentrated in 28 DPA *Gb* fiber, as compared to 21 DPA, whereas 5 of 23 were more concentrated in 28 DPA *Gh* fiber (Additional file 15). The upregulated metabolites at 28 DPA in both genotypes, including raffinose, were discussed previously. The five unique metabolites that were upregulated between 21 to 28 DPA in *Gb* fiber were adenosine, guanosine 3’-monophosphate (3’-GMP), galactinol, stachyose, and phosphoethanolamine. Metabolites that were strongly upregulated (8–138 fold) in 28 DPA *Gb* vs *Gh* fiber included adenosine 5’-diphosphate (ADP), three flavonoids (leucocyanidin, catechin, gallocatechin), stachyose, glucarate, and ascorbate (Table 4). Phosphoethanolamine is a precursor for synthesis of the phospholipids required to support continuing fiber elongation. In addition to ascorbate and flavonoids, galactinol and raffinose can scavenge free radicals [102]. Stachyose is also within the family of ‘raffinose oligosaccharides’ and may play a similar role in ROS remediation in cotton fiber. These oligosaccharides may also act as osmolytes to increase turgor pressure during a prolonged elongation period [55]. Glucarate (or D-glucaro-1,4-lactone) is able to protect biomolecules, such as lipids and proteins, against oxidative damage by ROS, including H_2_O_2_ [103]. In summary, several ways of looking at the data implicate enhanced ROS management as a key feature supporting the prolonged elongation and greater final length of the fiber of *Gb* cv Phytogen 800 as compared to *Gh* cv Deltapine 90.

## Conclusions

Deep transcriptomic and metabolomic analyses provided unparalleled insights into similarities and differences of fiber development in two genotypes of commercial allotetraploid cotton with well-characterized fiber development profiles. The implications of this rich data set can be experimentally tested in numerous ways. As examples, the expression levels of putatively important transcripts can be manipulated, including analysis of the presence and activity of the encoded proteins. Attempts can be made to change the metabolome of short-fibered cotton to be more like this cultivar of *Gb* fiber. Cotton diversity panels can be analyzed to look for repetitive correlations between useful fiber traits and particular genes and metabolites implicated by our data.

The specific stages analyzed in this study included primary wall synthesis to support high-rate elongation, transitional cell wall remodeling, and secondary wall thickening via mainly cellulose synthesis. During high rate elongation at 10 DPA, the transcriptomes and metabolomes of the fibers of both genotypes were complex with about 88 % of the transcripts and 95 % of the metabolites analyzed in this study being detected. Transitional cell wall remodeling was a discrete, relatively stable developmental stage lasting at least four days (18 to 21 DPA). Transcription factors associated with secondary wall synthesis in sclerenchyma cells began to be expressed then and persisted until 28 DPA when cellulose synthesis dominates. However, numerous transcripts encoding lignification activators were missing, whereas transcripts encoding a lignin repressor were present at 28 DPA. Correspondingly, the monolignol precursors of lignin were not detected in the cotton fiber metabolome. Therefore, commercial cotton fiber is white, soft, and absorbent due to the repression of lignification at the transcriptional level.

The data consistently supported a greater capacity for managing ROS in long cotton fiber, which correlated with higher protein levels and less protein degradation in the *Gb* cultivar as compared to the *Gh* cultivar we analyzed. The data showed a greatly increased level of ascorbate during the late elongation phase of the fiber in *Gb* cv Phytogen 800, and transcriptional clues suggested this is likely due to the activity of particular GLOase isoforms. In ongoing experiments, we are testing our prediction that enhanced ascorbate biosynthesis and recycling contributes to the extended elongation period and longer final length of *Gb* fiber either through general ROS management, by indirect modification of cell wall polysaccharides, or both. Further mining of the data will point to many fruitful avenues for future experimentation on the genetic and biochemical regulators of cotton fiber morphogenesis and quality.

## Methods

### Plant growth

The cotton cultivars used in this study were *Gossypium hirsutum* cv Deltapine 90 and *Gossypium barbadense* cv Phytogen 800. Plants were grown in parallel at the North Carolina State University Phytotron in a controlled greenhouse with a 26 °C/22 °C day/night temperature cycle. Under these well-controlled conditions, the plants developed vigorously with similar vegetative growth. The temporal progression of fiber development was repeatable between growing cycles. As needed, 1000 W metal halide lights were used to supplement day length to approximately 12 h a day. Plants (one per 6-L pot) were potted in a 2:1 (v/v) mixture of washed and sterilized gravel (# 16, construction grade) and Redi-earth Plug and Seedling Mix (#F1153; Sun Gro Horticulture Canada; based on the original “Cornell Mix”, [104]. Plants were watered twice daily with a solution containing macro- and micronutrients [105]. Fourteen plants of each cotton genotype were arranged in 4 rows of 7 plants each on wheeled carts holding one plant from each genotype. The carts were rotated once per week to equalize exposure of each plant to light on the edges of the plant block. Samples combined into one RNA pool were selected from positions distributed throughout the plant block.

### Fiber sampling

Flowers were tagged upon opening, and fiber for each DPA sampled (10, 15, 18, 21, and 28 DPA) was collected from a 1st position boll between nodes 10 and 16 (low to middle on the plant) between September and January. Bolls were primarily collected from 12:00-4:00 pm to minimize potential expression variability associated with time of day. All bolls included in the samples had diameter in a range previously established as typical for that DPA (data not shown), indicating normal growth of the fruits containing the assayed fibers. Within 5 min after the boll was harvested, bolls were cut open with a razor blade and cotton seed and fiber were frozen in liquid nitrogen. The samples were gently ground under nitrogen in a mortar and pestle until the developing seeds and fibers had separated, then seeds were removed with forceps. The remaining cotton fiber was then ground to a fine powder and stored at −80 °C until RNA and metabolite extraction.

### Overview of the statistical and gene identifier framework

Methods for statistical analysis are described in pertinent sections below. In the data interpretations, we generally highlighted significant differences defined for: (a) transcriptomics, q-values ≤ 0.001 and fold changes ≥ 2; and (b) metabolomics, *p*-values ≤ 0.05 and q-values ≤ 0.1, which indicates that ≤10 % of the discoveries called significant are possibly false positives. The q-values associated with *p*-values ≤ 0.05 in the metabolomics data are often considerably lower than 0.1 and both values are available in the supporting data. Arabidopsis gene identifiers, names and associated information are available at TAIR (http://www.arabidopsis.org/), and *Gr* gene identifiers and associated information are available at (www.phytozome.net). The heat maps and discussion generally relate only to primary transcripts (designated as such by read abundance in *Gr*, [47]), but data for all transcripts from one locus are available in the additional files.

### Transcriptomics

As detailed below, the transcriptome from each of 10 samples (5 DPA × 2 genotypes) was derived from three cDNA sequencing libraries, and each library contained RNA from the fiber of three bolls collected from different plants. Therefore, the transcriptome of each sample cumulatively represents fiber from three biological replicates inclusive of nine bolls from nine different plants. As described in detail below, > 2 billion reads were compiled from 37–97 M reads from each of 30 libraries (3 libraries × 5 DPA × 2 genotypes).

#### RNA extraction and quality control

RNA was extracted from 100–250 μl volumes of ground cotton fiber using a kit (Sigma Spectrum Total Plant RNA Kit; Sigma-Aldrich, www.sigmaaldrich.com) per the manufacturer’s instructions for protocol A and including an on-column DNAse digestion (Sigma DNase70 Kit; Sigma-Aldrich, www.sigmaaldrich.com). RNA quality was first assessed by spectrophotometry (Nanodrop; Thermo Scientific, www.thermoscientific.com) to ensure that all samples had a 260/280 absorbance between 2.0–2.2. Prior to library preparation RNA pool quality was assessed on the Agilent bioanalyzer (www.agilent.com).

#### Library preparation

For each sample, three cDNA libraries were prepared fee-for-service by the Genomic Sciences Laboratory at North Carolina State University (research.ncsu.edu/gsl/) using the Illumina TruSeq RNA sample prep kit v2 (www.illumina.com). Total RNA (1 μg) was purified using poly-T oligo-attached magnetic beads to isolate poly-A containing mRNA. The mRNA was chemically fragmented using divalent cations and fragments were reverse transcribed into first strand cDNA using random primers. Second strand synthesis was carried out using DNA Polymerase I and RNaseH. Ends were repaired on the double-stranded cDNA to convert overhangs into blunt ends, 3’ ends were adenylated, and adapters with indexes for multiplexing were ligated. The library was amplified with a PCR reaction and then small fragments were removed (AmpureXP beads; www.beckmancoulter.com). The final library was quantified on the Agilent Bioanalyzer High Sensitivity DNA Chip; (www.agilent.com) prior to pooling.

#### Sequencing

Six libraries per timepoint (3 per genotype) were pooled at equimolar concentration, denatured, diluted with hybridization buffer, and clustered at 9.5pM concentration via cBot in one lane of an Illumina TruSeq PE v3 flowcell. A PhiX v3 control library spike-in was included in the lane to monitor clustering and error rate during sequencing. The flowcell was sequenced on a HiSeq2000 instrument with TruSeq SBS v3-HS chemistry (Illumina; www.illumina.com) following the manufacturer’s instructions for 2 ×100 bp reads with single indexing.

#### Mapping reference and annotation

To generate a mapping reference, we combined information from *Gr* (D genome) and *Ga* (A genome) cotton, representative of the diploid ancestral genomes of modern tetraploid cotton. *Gr* transcripts were obtained from the D genome resource at PHYTOZOME (http://www.phytozome.net/) [47], and the assembled *Ga* ESTs were described in [48]. The *Gr* genome contains 37,505 protein coding genes and 77,267 annotated protein coding transcripts. Blastn was used to identify redundant sequences between the *Gr* transcripts and the *Ga* ESTs, and all sequences with ≥ E-10 match to a *Gr* transcript were omitted from the *Ga* contig assemblies in order to give priority to *Gr* genome information. A total of 98,807 cotton fiber transcripts generated in the current study were mapped to the reference, with 98 % of them matching *Gr* sequences. Note that this does not indicate an allelic expression bias. Instead it indicates our use of the *Gr* genome resources for further analysis whenever possible. This is a valid approach as indicated by only 1.8 % average amino acid difference between A and D protein orthologs [106].

Annotation information, including gene IDs for Arabidopsis homologs, was adopted from the cotton D genome resource at www.phytozome.net. These annotations were validated using Blastp against the TAIR peptide database (updated 12/14/2010). Of the RNA-Seq reads that we analyzed further (RPKM ≥ 2), only 2,106 of 41,566 reads (5 %) mapped uniquely to the *Ga* EST assemblies (NCBI Transcriptome Shotgun Archive (TSA) accession number GBYK00000000), and these were annotated if possible using Blastx against TAIR with 1E-5 cut-off. Additional sequence annotation and KEGG (www.genome.jp) reference pathway mapping was performed using Blast2GO version 2.7.2 (www.blast2go.com) [107]. Blastx was used to compare sequences against the NCBI non-redundant database with an expectation value cut-off of 1E-10 for sequences mapping to the *Gr* reference and 1E-4 for A genome contig assemblies. GO annotation was performed using Blast2GO default settings (1E-5) and was refined using the Blast2GO ANNEX tool.

#### Quantification of raw reads

Raw reads from the two genotypes of cotton fiber were mapped to the reference set using Burrows-Wheeler Aligner (BWA version 0.6.2; http://bio-bwa.sourceforge.net/) allowing for a maximum of 4 % mismatches. Sequence reads mapping to the reference were counted through BEDTOOLS (version 2.16.2; https://github.com/arq5x/bedtools2) after file format conversion using SAMTOOLS (version 0.1.18; http://samtools.sourceforge.net/). For normalization of read counts, RPKM values were calculated on the three replicates of each sample as: number of mapped reads per gene divided by total mapped reads and transcript size, followed by multiplication by 1 million. RPKM values for each gene were averaged from replicates for further analysis.

#### Differential gene expression

Differential gene expression was compared in a pair-wise manner within and between fiber genotypes. For *Gh* and *Gb* fiber, successive DPA were compared and the 10 vs 28 DPA comparison was included to emphasize the contrast between primary and secondary wall synthesis. For the between genotype comparisons, the data from the same DPA were compared in *Gh* vs *Gb* fiber. For differential gene expression, three biological replicates per sample were then subject to statistical tests, using the samWrapper package (DEGseq version 2.6; http://bioconductor.org/packages/release/bioc/html/DEGseq.html) in R (www.**r**-project.org) followed by multiple correction (FDR ≤ 0.001). The computational analysis of differential gene expression was performed in a Linux environment at TACC (Texas Advanced Computation Center) at University of Texas at Austin.

### Metabolomics

Five biological samples of 100 mg ground fiber were processed for each DPA. For 44 of the 50 samples, the same material used for sequencing was also used for metabolomic analysis (Metabolon Inc., Durham NC; www.metabolon.com). In a few cases, additional fiber was collected for metabolomics: 2 *Gb* bolls at 10 DPA and 1 boll each for *Gb* and *Gh* fiber at 18 and 21 DPA. Normalization was based on fiber dry weight, with a ratio of 15 μl extraction solvent per 1 mg. Protein was not used for data normalization due to the decline in total protein content (analyzed by Bradford assay) during fiber development and the higher protein level in *Gb* fiber (Fig. 4). Due to the approximately two-fold increase in the fiber weight per unit length ratio in both *Gh* and *Gb* fiber between 21 and 28 DPA [10], the 28 DPA samples were presumed to have less cytoplasmic contribution to the metabolite pool. Therefore, in the 28 DPA samples and within the 2-fold range, metabolite increases may be under-estimated and metabolite decreases may be over-estimated in terms of actual cytoplasmic concentration.

An automated sample preparation process was used (MicroLab STAR® system; hamiltonrobotics.com). For quality control, recovery standards were added prior to the first step in an aqueous and organic extraction process designed to remove proteins while maximizing recovery of small molecules. The resulting extract of each sample was divided into two aliquots for analysis by Liquid and Gas chromatography/mass spectrometry (GC/MS; LC/MS; LC/MS^2^). Residual organic solvent was evaporated (TurboVap®/Zymark; www.perkinelmer.com), and then each sample was frozen and dried under vacuum. The LC/MS and GC/MS methods were described previously [108, 109]. For GC/MS the column was 5 % phenyl and the temperature was ramped from 40° to 300 °C over 16 min. LC/MS and GC/MS data were automatically matched to a library of chemical standards as described in [110]. Compounds were identified as described previously [111, 112], but for our analysis a chemical library based on approximately 3000 standards was used. Normalization for instrument inter-day tuning differences, data scaling, and imputation of missing values were performed as described in [113].

### Analysis using R ver 3.1.1

Overrepresentation of KEGG pathways was calculated using the R function phyper and the family-wise error rate and false discovery rates were calculated using the R function p.adjust. Heat maps were produced on transcripts with RPKM ≥ 2 in either fiber genotype using the R function heat map.2 in the gplots package version 2.14.2 [114]. Due to display size limitations, alternative transcripts (as referenced to the *Gr* genome; www.phytozome.net; [47] were omitted from the heat map datasets, with the exception of the respiratory oxidative burst genes. This omission was done after checking that the correlation between primary and secondary transcripts from the same locus was ≥0.73 and that the annotations at Phytozome were identical. The only exception to this high correlation rule was for an alternative transcript related to chalcone synthase that was expressed in *Gh* fiber (R = 0.99 compared to the primary transcript), but the corresponding locus had essentially no expression in *Gb* fiber (RPKM < 0.14). Plots were generated using the R function barplot in the gplots package. Cluster analysis was done using the R package Mfuzz [49]. The optimal fuzzifier values were calculated for each set of transcripts and metabolites using the function mestimate. Potential optimal cluster numbers were estimated using the minimum centroid distance with the function dmin. Venn diagrams were drawn using the package VennDiagram [115].

### Availability of supporting data

The raw sequencing data supporting the results of this article have been deposited to the NCBI sequence read archive (http://www.ncbi.nlm.nih.gov/sra/) under accession SRP049330 and bioproject archive (http://www.ncbi.nlm.nih.gov/bioproject/) PRJNA263926. The contig assemblies from *G. arboreum* were deposited in the NCBI Transcriptome Shotgun Archive (TSA) (http://www.ncbi.nlm.nih.gov/genbank/tsa) under accession GBYK00000000. The processed data sets supporting the results of this article are included within the article and its additional files.

## Additional files

Additional file 1:
***Gb*** **Transcriptome data.** Transcript levels (RPKM) for *Gb* fiber at 10, 15, 18, 21, and 28 DPA. Additional file 2:
***Gh*** **Transcriptome data.** Transcript levels (RPKM) for *Gh* fiber at 10, 15, 18, 21, and 28 DPA. Additional file 3:
**Metabolome data.** Metabolite levels and the results of pair-wise statistical comparisons for *Gb* and *Gh* fiber at 10, 15, 18, 21, and 28 DPA. Additional file 4:
**KEGG pathway enrichment_Metabolites.** Overrepresented KEGG pathways in the fiber metabolome. Additional file 5:
**Blast2GO sequence annotation.** Top NCBI BLASTX hits for reference sequences with RPKM ≥ 2. Additional file 6:
**Blast2GO KEGG pathway mapping.** Predicted KEGG pathways and enzyme codes for reference sequences with RPKM ≥ 2. Additional file 7:
**KEGG pathway enrichment_Transcripts.** Overrepresented KEGG pathways as inferred from the combined fiber transcriptome from *Gb* and *Gh* fiber. Additional file 8:
**GO Biological Processes 28 vs 21 DPA.** Gene Ontology biological processes that are overrepresented in 28 DPA transcripts compared to 21 DPA in *Gb* or *Gh* fiber. Additional file 9:
***Gb*** **differentially expressed genes across DPA.** Transcripts differentially expressed for pairwise comparisons of *Gb* fiber at 10, 15, 18, 21, and 28 DPA. Additional file 10:
***Gh*** **differentially expressed genes across DPA.** Transcripts differentially expressed for pairwise comparisons of *Gh* fiber at 10, 15, 18, 21, and 28 DPA. Additional file 11:
**Transcript clusters.** The six transcript clusters in *Gb* or *Gh* fiber and the overrepresented GO biological processes associated with each cluster. Additional file 12:
***Gb*** **differentially expressed genes across genotypes.** Transcripts more highly expressed in *Gb* fiber compared to *Gh* fiber at 10, 15, 18, 21, and 28 DPA. Additional file 13:
***Gh*** **differentially expressed genes across genotypes.** Transcripts more highly expressed in *Gh* fiber compared to *Gb* fiber at 10, 15, 18, 21, and 28 DPA. Additional file 14:
**Cross-genotype GO Biological Processes enrichment.** Gene Ontology biological processes overrepresented in transcripts differentially expressed between *Gb* and *Gh* fiber. Additional file 15:
**High concentration and high change metabolites.** Tables of the most concentrated metabolites in *Gb* or *Gh* fiber and those with a 10 fold or greater change between 10 and 28 DPA. Additional file 16:
**Metabolite clusters.** Lists of metabolites in each of the four metabolite clusters found in *Gb* or *Gh* fiber. Additional file 17:
**TAIR blastp.** Homology and annotation information derived from TAIR Blastp for transcripts displayed in the heat maps and graphs. Additional file 18:
**Transcripts associated with respiratory oxidative burst.** Heat map of transcripts encoding Rho-like GTPases (RAC), respiratory burst oxidase homologs (RBOH), and copper and manganese superoxide dismutase (CSD, MSD). Additional file 19:
**Comparing RNA-Seq and Microarray Data.**
Additional file 20:
**Transcripts for extended elongation.** A list of 1288 transcripts that potentially contribute to extended elongation in *Gb* fiber at 28 DPA. Additional file 21:
**Extended elongation GO Biological Processes.** Gene Ontology biological processes overrepresented in the 1288 transcripts correlated with extended elongation in *Gb* fiber at 28 DPA.
