# Supplementary figures and images for: Metabolomic and transcriptomic insights into how cotton fiber transitions to secondary wall synthesis, represses lignification, and prolongs elongation
Source: BMC Genomics. 2015 Jun 27;16(1):477. doi: 10.1186/s12864-015-1708-9 (PMC4482290; doi:10.1186/s12864-015-1708-9)

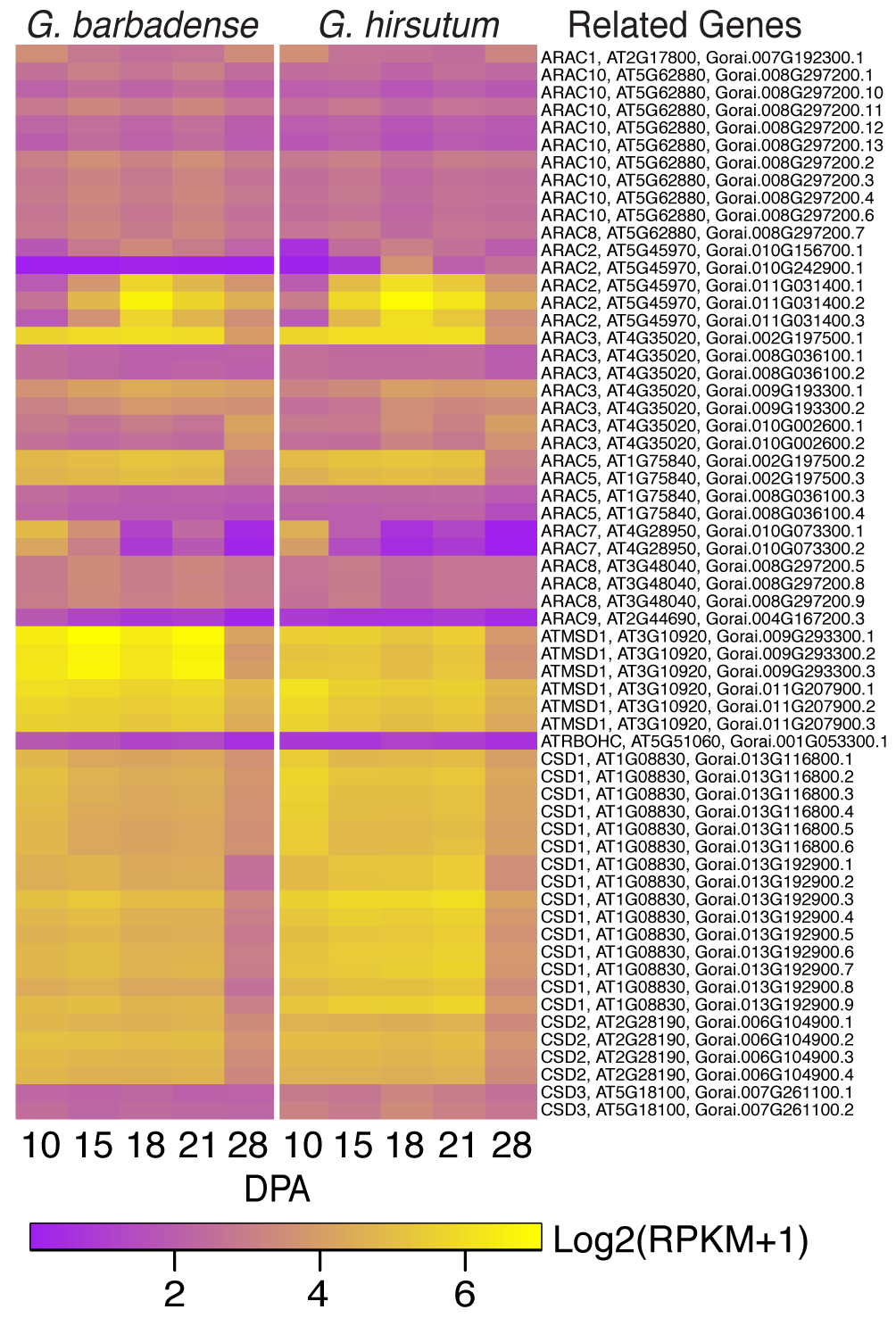

Supplement: Additional file 18: — Transcripts associated with respiratory oxidative burst. Heat map of transcripts encoding Rho-like GTPases (RAC), respiratory burst oxidase homologs (RBOH), and copper and manganese superoxide dismutase (CSD, MSD). [file 12864_2015_1708_MOESM18_ESM.tiff]
