# Supplementary material for: Metabolomic and transcriptomic insights into how cotton fiber transitions to secondary wall synthesis, represses lignification, and prolongs elongation
Source: BMC Genomics. 2015 Jun 27;16(1):477. doi: 10.1186/s12864-015-1708-9 (PMC4482290; doi:10.1186/s12864-015-1708-9)
Supplement: Additional file 19: — Comparing RNA-Seq and Microarray Data. [file 12864_2015_1708_MOESM19_ESM.pdf]

Additional File 19
Comparing RNA-Seq and Microarray Data

Metabolomic and transcriptomic insights into how cotton fiber transitions to secondary wall synthesis, represses lignification, and prolongs elongation

John R. Tuttle, Gyoungju Nah, Mary V. Duke, Danny C. Alexander, Xueying Guan, Qingxin Song, Z. Jeffrey Chen, Brian E. Scheffler, Candace H. Haigler

To explore whether the gene encoding gulono-1,4-lactone oxidase (called GulLO6 or GLOase) was up-regulated in other *Gh/Gb* cotton fiber comparisons, we analyzed published microarray data. Previously, gene expression in another pair of cultivars, *Gh* cv TM-1 and *Gb* cv Hai7124, was compared at similar days post anthesis (DPA) using a microarray platform [1]. The data from the prior study are available under NCBI GEO accessions GPL2610, GPL8569, and GSE18028.

As a basis for our search, we used 288 differentially expressed genes at 10 or 28 DPA that were highlighted in the main text of Tuttle et al. describing the RNA-Seq data. We checked the correspondence of these 288 genes with 415 genes that were called differentially expressed at 10 or 25 DPA in the published microarray paper [Table S1 of reference 1]. Note that in the microarray study [1] only 10 and 25 DPA data were used to evaluate differential expression, although data were available in the primary data files for 10, 15, 20, and 25 DPA as shown in Figure S1. The time-points of 25 and 28 DPA are likely to represent similar physiological times in fiber development (secondary wall cellulose synthesis stage, with continuing elongation only in *Gb* fiber) despite the differences in growing conditions in the two studies. The similar patterns of gene expression shown in Figure S1 below support the similar timing of fiber development in the two studies.

Blastn was used to match the relevant *G. raimondii* transcript identifiers (Additional file 17) with the most homologous microarray probes. E-values are displayed in Table S1 below. Sequences for the subset of genes implicated as being differentially expressed by RNA-Seq were downloaded from Phytozome and compared to the microarray probe sequences using blastn (1E-5 cut-off) to obtain microarray probe IDs with high homology to *G. raimondii* transcripts. To produce the comparative heatmap (Figure S1), *Gh/Gb* untransformed microarray ratios from [1] were downloaded from NCBI GEO accession number GSE18028, averaged, and log2 transformed for comparison to log2 transformed RPKM ratios derived from the RNA-Seq data in Tuttle et al. Table S1 below shows the raw data used to produce the heat map.

Five microarray probes were homologous to the same *G. raimondii* identifiers as differentially expressed genes in the subset of the RNA-Seq data used in this comparison. Based on high E-values as compared to Arabidopsis genes (Additional File 17), these are expected to encoded enzymes involved in synthesis of xyloglucan (CSLC4, cellulose-synthase-like C4), lignin (HCT, hydroxycinnamoyl-coA/Shikimate hydroxycinnamoyl-transferase), flavonoids (CHS, chalcone synthase), and ascorbate (GulLO6/GLOase). The *Gh/Gb* expression ratios had similar patterns in the RNA-Seq and the microarray data for each comparison. Specifically, *Gh* fiber had higher expression of *CSLC4* and two *CHS* isoforms, whereas *Gb* fiber had higher expression of *HCT* early in fiber development and *GulLO6* (GLOase) later in fiber development.

The authors of [1] did not discuss the *GulLO6* (GLOase) gene expression data in their manuscript, probably because the expression difference between the cultivars was significant but relatively low, as may often happen due to the lower dynamic range of microarray signals as

compared to RNA-Seq. Alternatively, the expression bias in *Gb* cv Hai7124 as compared to *Gh* cv TM-1 might be of lesser magnitude than in the two cultivars compared in Tuttle et al. The important point is that two independent comparisons showed up-regulated *GulLO6* (*GLOase*) gene expression in *Gb* cotton fiber. The similar expression patterns shown for the other genes in the heat map (Figure S1) supports the general reliability of the RNA-Seq data.

Reference

[1] Chen X, Guo W, Liu B, Zhang Y, Song X, Cheng Y, Zhang L, Zhang T: **Molecular mechanisms of fiber differential development between *G. barbadense* and *G. hirsutum* revealed by genetical genomics.** *PLOS ONE* 2012, **7**(1):DOI:10.1371/journal.pone.0030056.

Table S1. Comparison of a sub-set of genes from the RNA-Seq experiment in Tuttle et al with a published microarray experiment on another pairwise comparison of *Gh* and *Gb* cultivars [1].

RNA-Seq Sub-set of Differentially Expressed Genes							Homologous GPL2610 Microarray Differentially Expressed Genes					
REFERENCE ID	TAIR LOCUS	10 DPA	15 DPA	20 DPA	28 DPA	GENE NAME	GPL2610 ID	10 DPA	15 DPA	20 DPA	25 DPA	E-value
Gorai.006G028400.1	AT3G28180	1.84	1.20	1.33	0.09	CSLC4	CM043G11	0.62	0.40	-0.02	0.56	0
Gorai.006G028400.2	AT3G28180	1.85	1.15	1.26	0.05	CSLC4	CM043G11	0.62	0.40	-0.02	0.56	0
Gorai.006G028400.3	AT3G28180	1.83	1.17	1.23	0.07	CSLC4	CM043G11	0.62	0.40	-0.02	0.56	0
Gorai.011G130700.1	AT5G48930	-2.30	-1.39	-1.06	-0.03	HCT	CM048C05	-2.06	-0.23	0.02	0.33	0
Gorai.011G130700.2	AT5G48930	-2.72	-1.72	-1.25	0.00	HCT	CM048C05	-2.06	-0.23	0.02	0.33	0
Gorai.011G130700.3	AT5G48930	-2.20	-1.35	-1.12	0.00	HCT	CM048C05	-2.06	-0.23	0.02	0.33	0
Gorai.011G130700.4	AT5G48930	-2.74	-1.74	-1.32	-0.03	HCT	CM048C05	-2.06	-0.23	0.02	0.33	0
Gorai.011G130700.5	AT5G48930	-2.75	-1.76	-1.26	-0.03	HCT	CM048C05	-2.06	-0.23	0.02	0.33	0
Gorai.011G130900.1	AT5G48930	-2.66	-0.79	-0.47	-0.01	HCT	CM048C05	-2.06	-0.23	0.02	0.33	5E-154
Gorai.011G131500.1	AT5G48930	-5.26	-3.23	-2.54	-0.10	HCT	CM048C05	-2.06	-0.23	0.02	0.33	0
Gorai.011G132100.1	AT5G48930	-6.11	-4.52	-3.92	-0.23	HCT	CM048C05	-2.06	-0.23	0.02	0.33	0
Gorai.006G000200.1	AT5G13930	5.41	4.90	4.53	5.75	CHS	CM067B03	1.78	2.60	1.83	1.04	5E-125
Gorai.006G000200.2	AT5G13930	5.14	4.93	4.45	5.46	CHS	CM067B03	1.78	2.60	1.83	1.04	2E-113
Gorai.005G035100.1	AT5G13930	5.05	3.42	4.35	4.30	CHS	CM100D01	1.29	1.84	1.69	1.18	0
Gorai.008G129900.1	AT2G46760	-0.55	-5.88	-6.75	-5.57	GulLO6	CM047E10	-0.18	-1.26	-0.61	-0.92	0
Gorai.008G130100.1	AT2G46760	-1.29	-4.61	-5.63	-4.34	GulLO6	CM047E10	-0.18	-1.26	-0.61	-0.92	0

Gh/Gb expression ratios are shown.
RNA-Seq values are LOG2(Gh RPKM+1/Gb RPKM+1)
GPL2610 values are LOG2(Gh/Gb Lowess-normalized microarray signal intensities from NCBI GEO accession GSE18028)
E-values are for blastn (E-value ≤ 1E-5) comparisons of *G. raimondii* reference sequences against microarray probe sequences.

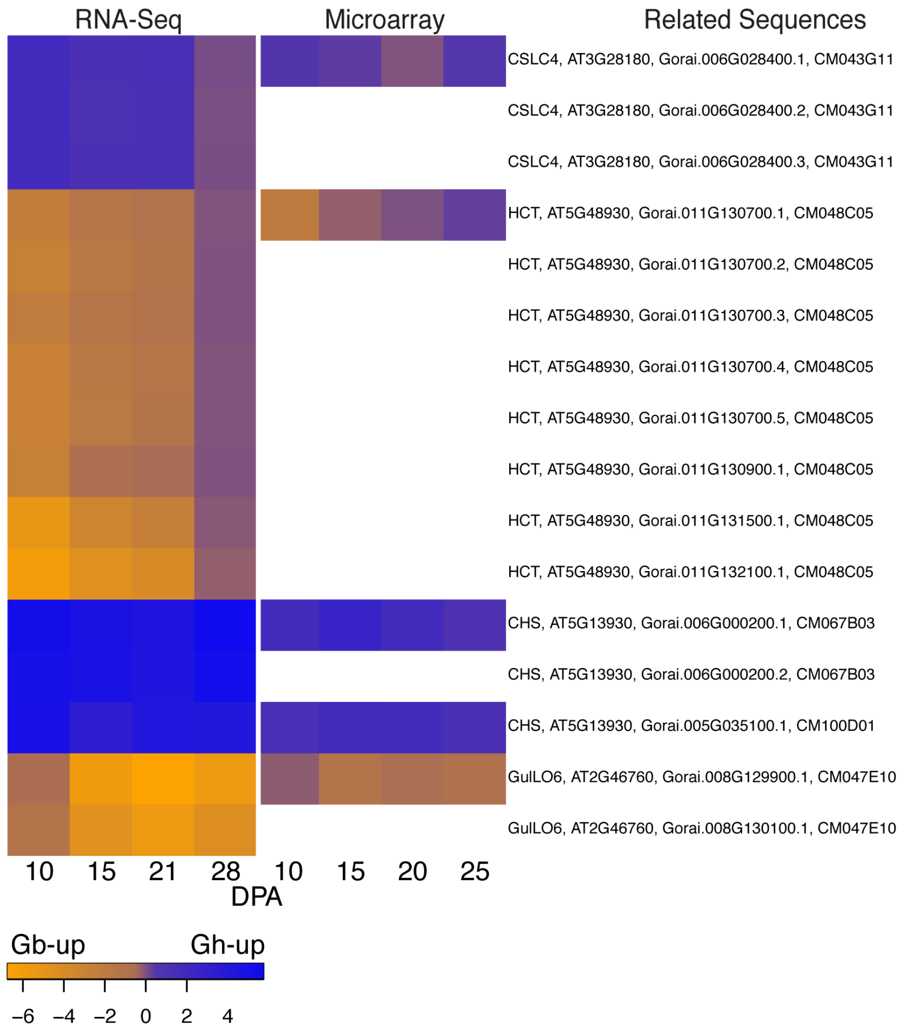

Figure S1. Heat Map showing that the expression of *GulLO6 (GLOase)* is up-regulated in a published microarray experiment comparing two other cultivars of *Gh* and *Gb* cotton [1]. This heat map shows the ratios of *Gh/Gb* gene expression for selected genes that arose through the comparative strategy described above, including *GulLO6 (GLOase)*. Higher expression in *Gb* and *Gh* cotton fiber is indicated by orange and blue, respectively. The values used to create the heatmap were log2-transformed *Gh/Gb* ratios of average RPKM plus one (RNA-Seq) or Lowess-normalized *Gh/Gb* signal intensity ratios (microarray) (see Table S1). The related sequences include the gene name and identifier number for the Arabidopsis homolog, the *G. raimondii* transcript identifier homologous to the *Gh* and *Gb* sequences, and the 'CM' identifiers of the probes on the GPL2610 array.
